# SIRT1 inhibits mitochondrial hyperfusion associated mito-bulb formation to sensitize oral cancer cells for apoptosis in a mtROS-dependent signalling pathway

**DOI:** 10.1038/s41419-023-06232-x

**Published:** 2023-11-10

**Authors:** Srimanta Patra, Amruta Singh, Prakash P. Praharaj, Nitish K. Mohanta, Mrutyunjay Jena, Birija S. Patro, Ali Abusharha, Shankargouda Patil, Sujit K. Bhutia

**Affiliations:** 1 Cancer and Cell Death Laboratory, Department of Life Science, National Institute of Technology, Rourkela, Odisha 769008 India; 2 Post Graduate Department of Botany, Berhampur University, Bhanja Bihar, Berhampur, 760007 India; 3 Bio-Organic Division, Bhabha Atomic Research Centre, Mumbai, 400085 India; 4 Optometry Department, Applied Medical Sciences Collage, King Saud University, Riyadh, 145111 Saudi Arabia; 5 College of Dental Medicine, Roseman University of Health Sciences, South Jordan, 84095 UT USA; 6 Centre of Molecular Medicine and Diagnostics (COMManD), Saveetha Dental College & Hospitals, Saveetha Institute of Medical and Technical Sciences, Saveetha University, Chennai, 600 077 India

**Keywords:** Oral cancer, Cell death

## Abstract

SIRT1 (NAD-dependent protein deacetylase sirtuin-1), a class III histone deacetylase acting as a tumor suppressor gene, is downregulated in oral cancer cells. Non-apoptotic doses of cisplatin (CDDP) downregulate SIRT1 expression advocating the mechanism of drug resistance. SIRT1 downregulation orchestrates inhibition of DNM1L-mediated mitochondrial fission, subsequently leading to the formation of hyperfused mitochondrial networks. The hyperfused mitochondrial networks preserve the release of cytochrome C (CYCS) by stabilizing the mitochondrial inner membrane cristae (formation of mitochondrial nucleoid clustering mimicking mito-bulb like structures) and reducing the generation of mitochondrial superoxide to inhibit apoptosis. Overexpression of SIRT1 reverses the mitochondrial hyperfusion by initiating DNM1L-regulated mitochondrial fission. In the overexpressed cells, inhibition of mitochondrial hyperfusion and nucleoid clustering (mito-bulbs) facilitates the cytoplasmic release of CYCS along with an enhanced generation of mitochondrial superoxide for the subsequent induction of apoptosis. Further, low-dose priming with gallic acid (GA), a bio-active SIRT1 activator, nullifies CDDP-mediated apoptosis inhibition by suppressing mitochondrial hyperfusion. In this setting, SIRT1 knockdown hinders apoptosis activation in GA-primed oral cancer cells. Similarly, SIRT1 overexpression in the CDDP resistance oral cancer-derived polyploid giant cancer cells (PGCCs) re-sensitizes the cells to apoptosis. Interestingly, synergistically treated with CDDP, GA induces apoptosis in the PGCCs by inhibiting mitochondrial hyperfusion.

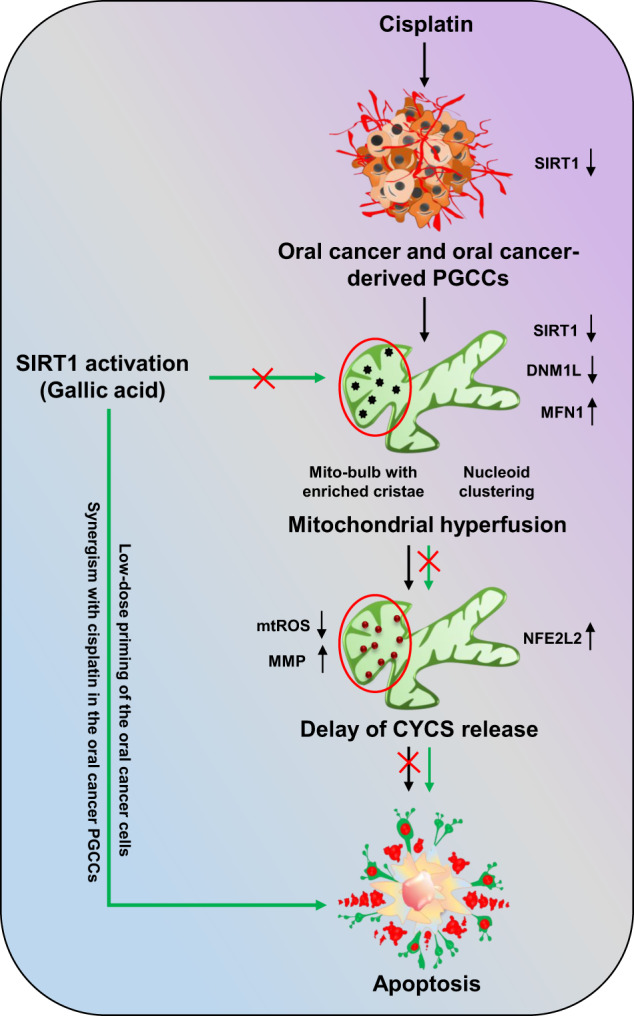

## Introduction

Mitochondria, the highly dynamic cellular organelles, are the chief architects for maintaining cellular homeostasis via fusion and fission cycles [1–3]. DNM1L/DRP1 critically regulates mitochondrial fission as it makes a constriction site on the mitochondria where the adaptor proteins teether and enable mitochondrial fission. During external stimuli, the mitochondrial fission gets hyperactivated or inhibited, resulting in cell survival or death. Inhibition of DNM1L mediated mitochondrial fission grades hyperfused mitochondrial network that physiologically supports cell survival under stress [2, 4, 5]. The mitochondrial transmembrane GTPases MFN1 and MFN2, along with dynamin-related GTPase; OPA1, facilitate mitochondrial hyperfusion [6]. Due to the enlarged branch length, the hyperfused mitochondria are expected to be safe from mitophagic clearance [7]. It has also been delineated that exposure to non-apoptotic stimuli directs mitochondrial hyperfusion via inhibiting mitochondrial fission as an oxidative stress-mitigating mechanism to guard the cells against apoptosis [8]. Moreover, the hyperfused mitochondrial networks entrap mitochondrial DNA (mtDNA) and cytochrome C (CYCS) in their mito-bulb like nucleoid clusters to safeguard the cells against apoptosis. The mtDNA dynamics have shown that tightly packed mtDNA division has been closely associated with mitochondrial fission [9]. In mitochondrial fission defective cells, the mtDNA is stacked in the mitochondrial inner membrane (IM), possibly restricting mtDNA transfer to the defective offspring [9, 10]. However, mtDNA maintenance under mitochondrial dynamics is still elusive.

Oral squamous cell carcinoma, a subtype of head and neck squamous cell carcinoma, has a higher prevalence and reoccurrence due to effective genetic modification of oncogenes associated with it [11, 12]. Epigenetic modifications play a propulsive role in the malignant transformation of oral cancer, leading to drug resistance [13]. SIRT1, a NAD-dependent class III histone deacetylase, has a dual role in cancer [14]. Overexpression of SIRT1 has been associated with the malignant transformation of several cancer subtypes, while oral cancer and glioblastoma cells exhibit minimal expression of SIRT1 [15–18]. In oral cancer cells, SIRT1 is found to be downregulated [16]. As an upstream target of DNM1L, SIRT1 regulates the mitochondrial dynamics for cancer cell survival during chemotherapeutic stress. SIRT1 downregulation redirects mitochondrial hyperfusion by inhibiting DNM1L-mediated mitochondrial fission [19, 20]. Hence, inhibition of mitochondrial hyperfusion by SIRT1 activation could trigger mitochondrial membrane permeabilization and mitochondrial superoxide generation, which acts as precursors for apoptosis induction. Previously, it has been demonstrated that SIRT1-activating drugs have immense potential as anticancer drugs, hence targeting SIRT1 might give a novel therapeutic avenue for treating oral cancer [17].

In this study, we have observed that SIRT1 activation led to DNM1L phosphorylation which subsequently controlled mitochondrial hyperfusion to trigger apoptosis in oral cancer cells. SIRT1-mediated DNM1L activation facilitated formation of DNM1L-BAX complex at the mitochondrial outer membrane (OM) for subsequent pore formation. Moreover, the re-introduction of SIRT1 regulated mtDNA dynamics and distribution, which is critical for apoptosis induction. Our study also showed that hyperfusion inhibits mitochondrial superoxide generation and regulates NFE2L2 and antioxidant enzymes to modulate apoptosis. Moreover, SIRT1 activation potently induces cell death by apoptosis in oral cancer and cisplatin (CDDP)-resistance oral cancer cells via inhibiting mitochondrial hyperfusion. Interestingly, we also identified a bioactive SIRT1 activator, gallic acid (GA), that inhibits mitochondrial hyperfusion leading to mitochondrial superoxide generation and apoptosis. In addition, synergistic treatment of GA and CDDP is effective against CDDP-resistant oral cancer cells by re-sensitizing them to apoptosis.

## Materials and methods

### Cell culture, generation of polyploid giant cancer cells and GA-primed cells

Oral cancer cell lines such as UMSCC-22A, UMSCC-22B, Cal27, Cal33 and FaDu were procured from the American Type Culture Collection, USA and German Collection of Microorganisms and Cell Cultures, Germany. Cervical cancer cell line HeLa; colorectal cancer cell line HT-29; prostate cancer cell lines DU145 and PC3 and breast cancer cell lines MDA-MB-231 and MCF-7 were procured from National Center for Cell Science, Pune, India. All the cell lines were cultured in Dulbecco’s Modified Eagle’s Medium with high glucose with 10% fetal bovine serum and 1% antibiotic–antimycotic solution under 5% CO_2_ at 37 °C.

At 70–80%, confluent oral cancer cells (Cal33 and FaDu) were treated with 15 µM CDDP (Sigma-Aldrich, P4394) for 10 h. The cells were allowed to recover for 7 days in a regular DMEM medium (the media was replaced every 2 days). The polyploid giant cancer cells (PGCCs) were visualized to determine their characteristics and then subcultured. The PGCC escapers/ daughters were visualized in the subsequent 15 days recovery period [21, 22].

For the generation of GA (Sigma-Aldrich, G7384; 2.5 µg/mL)-primed cells, low dose of GA (was treated to the Cal33 and FaDu cells for 21 days (the media was replaced every 3 days). After 21 days, the GA-primed cells were tested by MTT (Sigma-Aldrich, M5655) assay for their sensitivity towards GA and CDDP.

### Antiproliferative assay; cell viability and clonogenic assay

About 4 × 10^3^ cells/well were cultured in a 96-well plate, and different doses of CDDP and GA were treated in the cells according to the experimental requirements for 24 h. 5 mg/mL of MTT solution was added to the wells 4 h before termination of the experiment. Post-dark incubation, the optical density was measured at 595 nm.

After 10 h treatment of desired drugs with responsive concentrations, about 1000 cells/well were cultured in a 6-well plate for 2 weeks with media replaced every three days. After 14 days, the cells were fixed and stained with 0.5% crystal violet solution. Colonies with more than 45 cells were counted under a bright field microscope.

### Caspase 3/7 Glo assay for apoptosis detection

After the desired treatment, the protein isolates of the treated cells were subjected to the Caspase 3/7 Glo reagent following the manufacturer’s protocol (G8090, Promega Corp., Madison, WI, USA).

### Detection of mitochondrial membrane potential

The CDDP-treated cells were incubated in 10 µg/mL of Rhodamine 123 (Sigma-Aldrich, R8004) for 15 min in the dark. After washing with PBS, the cells were visualized under a confocal microscope (Leica TCS SP8).

### Analysis of mitochondrial superoxide generation

After treating with desired concentrations, the treated cells were incubated in 50 nM of MitoSOX (Thermo Fisher Scientific, M36008) for 30 min dark. After washing with PBS, the cells were subjected to flow cytometric analysis (BD Accuri C6™ flow cytometer). The mean FL2A intensity was normalized with unstained and control samples and represented as relative fold change in the fluorescence intensity.

### Enzymatic antioxidant assay

#### Estimation of SOD by enzymatic antioxidant assay

The enzymatic estimation of SOD in the whole cell lysates of the treated cells was done by following methods described by Weydert with a few modifications [23].

#### Estimation of CAT by enzymatic antioxidant assay

The enzymatic estimation of CAT in the whole cell lysates of the treated cells was done by following methods described by Weydert with a few modifications [23].

#### Estimation of APX by enzymatic antioxidant assay

After treatment, 50 μg of the isolated whole cell lysate was mixed with 0.5 mM ascorbate, 50 mM sodium phosphate buffer (pH 7) and 10 mM of H_2_O_2_. Spectrophotometric degradation of H_2_O_2_ was determined using the standard extinction coefficient to measure the APX activity [24].

#### Estimation of GR by enzymatic antioxidant assay

After treatment, 50 μg of the isolated whole cell lysate was mixed with 0.5 mM oxidized glutathione, 2 mM EDTA, 0.2 mM NADPH and 2 M tris-buffer (pH 7.8). The NADPH oxidation was spectrophotometrically measured at 340 nm to measure GR activity [24].

#### Estimation of GP_X_ by enzymatic antioxidant assay

The enzymatic estimation of GP_X_ in the whole cell lysates of the treated cells was done by following methods described by Weydert with few little modifications [23].

### Confocal imaging for live cell staining and co-localization studies

For mitochondrial branch length and skeleton analysis; MitoTracker Deep Red FM (Thermo Fisher Scientific, M22426; 30 nM, 15 min dark incubation), for mitochondrial nucleoids analysis; SYBR Green I (Thermo Fisher Scientific, S7563; 30 min dark incubation) were added to the cells after drug treatment and mitochondrial DNA synthesis inhibitor lamivudine (TCI Chemicals, L0217, 10 µM) treatment. The cells were counterstained with Hoechst 33342 (Sigma-Aldrich, B2261; 1 µg/mL, 5 min at RT) before visualization. For the co-localization studies, the cells were fixed, blocked and permeabilized before incubation of primary antibody (overnight at 4 °C) followed by secondary antibody incubation in Alexa Flour 488 (Thermo Fisher Scientific, A11001 and A11008) and Alexa Flour 568 (Thermo Fisher Scientific, A11004 and A11011), counterstained with DAPI (Sigma-Aldrich, D9542; 1 µg/mL, 5 min at RT) and visualization under confocal microscope.

### Western blot analysis

After desired treatment, the whole cell lysates were collected from the treated cells. An equivalent amount of proteins were subjected to SDS-PAGE followed by membrane transfer, primary antibody incubation, secondary antibody (BioBharati; secondary mouse; BB-SAB 02 C and secondary rabbit; BB-SAB 01 C) incubation and visualization by the chemiluminescence method. To check mitochondrial dynamics, the expression of SIRT1 (CST; #9475), p-DNM1L (CST; #3455), FIS1 (SantaCruz; sc-376469), MFN1 (SantaCruz; sc-166644) and MFN2 (SantaCruz; sc-515647) were analyzed by incubation in respective primary antibodies. For the antioxidant assay, the expression of NFE2L2 (SantaCruz; sc-722) was checked. TFAM (SantaCruz; sc-166965) was used to confirm the mtDNA in the nucleoids. ACTB (ABclonal; AC026) expression was considered as endogenous loading control for all the experiments. For subcellular fractionation assay, post drug treatment, the cells were harvested with trypsin-EDTA. Using NE-PER Nuclear and Cytoplasmic Extraction Reagents Kit (Thermo Fisher Scientific, 78835), the nuclear and cytoplasmic fractions were isolated following manufacturer’s protocols. COX4 (CST; #4844) and ACTB were used respectively as loading control for mitochondrial and cytoplasmic protein fractions.

### Meta-analysis and screening of potential SIRT1 activator

The available literature on PubMed, Google Scholar and other online sources were searched with keywords such as HDAC, SIRTs, SIRT activators, SIRT inhibitors, SIRT1, diseases, therapeutics, cancers and oral cancer. For the target identification in oral cancer, Schomberg’s article was followed thoroughly. The Cystoscape was used for the SIRT1-target protein interactions. The SIRT1 activator was identified by using in-house bio-active compound libraries. Further identification of ADME was done using the online tool SWISS ADME.

### Statistical analysis

All the experiments were done at least thrice as experimental replicates. The mean ± SD was evaluated with two-way ANOVA for samples with multiple variables. Student’s *t*-test was performed for samples with two variables. GraphPad Prism 9 (GraphPad Software, Inc., San Diego, CA, USA) was used for the analysis of all the statistical parameters. The defined *p*-value was found to be: *p*-value > 0.05 was considered not significant (ns), *p-value < 0.05, **p-value < 0.01, ***p-value < 0.001 and ****p-value < 0.0001; ^#^*p*-value was the comparison of treatment group with inhibitor-cotreated group with a similar level of significance. ^@^*p*-value was the comparison of the inhibitor group with inhibitor-cotreated group with a similar level of significance.

## Results

### SIRT1 downregulation is a detectable marker in oral cancer cells under basal and cisplatin resistance conditions

**Fig. 1:**
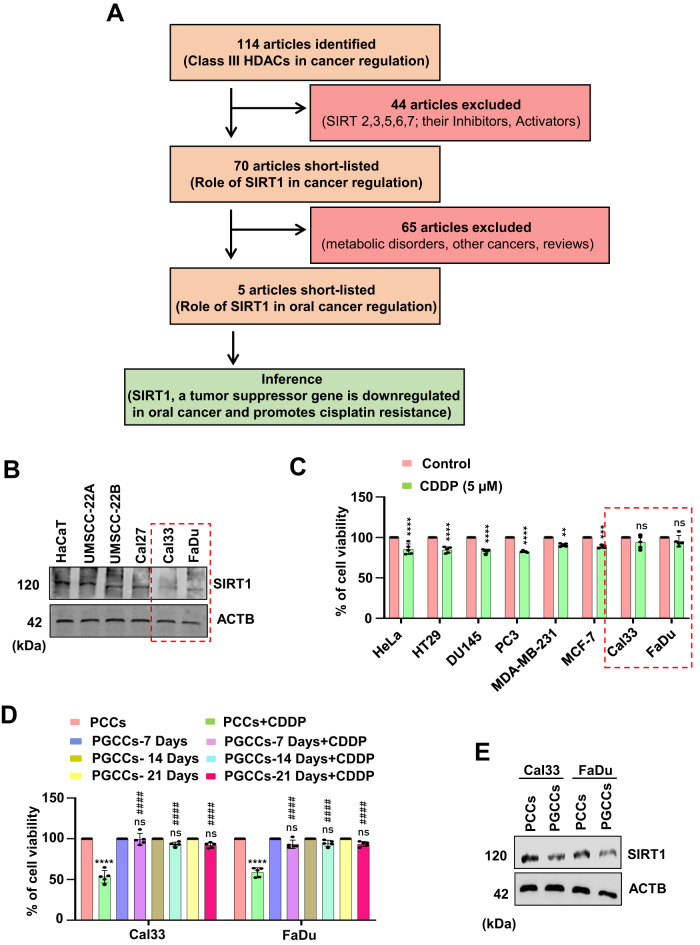
SIRT1 is downregulated in oral cancer and oral cancer-derived PGCCs. Schematic diagram of meta-analysis signifying the critical involvement of SIRT1 during malignant transformation of oral cancer (**A**). Analysis of SIRT1 expression across different oral cancer cell lines (**B**). Cell viability by MTT cytotoxicity in various cancer cell lines after CDDP treatment (**C**). Analysis of cell viability by MTT assay in oral cancer-derived PGCCs confirming CDDP resistance (**D**). Analysis of SIRT1 expression in oral cancer-derived PGCCs (**E**). Data represented in mean ± SD; students *t*-test for two variables and two-way ANOVA for multiple variables. The *p*-value > 0.05 was considered not significant (ns), *p-value < 0.05, **p-value < 0.01, ***p-value < 0.001 and ****p-value < 0.0001; ^#^*p*-value was the comparison of the treatment group with the inhibitor-cotreated group with a similar level of significance.

Aberrant post-translational modifications such as histone deacetylation fuels aggressive oral carcinogenesis and therapeutic resistance by manipulating various death pathway mediators. Here, we investigated the correlation between the expression of SIRTs and the malignant transformation of oral cancer. The meta-analysis study showed that SIRT1 was found to be downregulated in oral cancer cells under basal as well as CDDP resistance conditions (Fig. 1A). Interestingly, the expression of SIRT1 was found to be downregulated across oral cancer cell lines with minimal expression in Cal33 and FaDu cells (Fig. 1B and Supplementary Fig. S1A). Further, Cal33 and FaDu cells showed resistance against cisplatin as compared to different other cancer cell types (Fig. 1C). Further interaction of SIRT1 was validated with the top 5% influential genes that are expressed in platinum treated (Supplementary Fig. S1B) and platinum non-treated (Supplementary Fig. S1C) oral cancer tumors (influential genes are derived from the chemo-informatics analysis of Schomberg).

In addition, we confirmed the therapeutic resistance of cisplatin in oral cancer derived PGCCs (Fig. 1D and Supplementary Fig. S2A–F_ii_). Intriguingly, the PGCCs derived from Cal33 and FaDu cells had shown SIRT1 downregulation as compared to parent cancer cells (Fig. 1E and Supplementary Fig. S2G). Collectively, these results concluded that SIRT1 is downregulated in oral cancer cells under basal and cisplatin resistance conditions.

### SIRT1 downregulation potentiate mitochondrial hyperfusion by inhibiting mitochondrial fission with the treatment of non-apoptotic doses of cisplatin

**Fig. 2:**
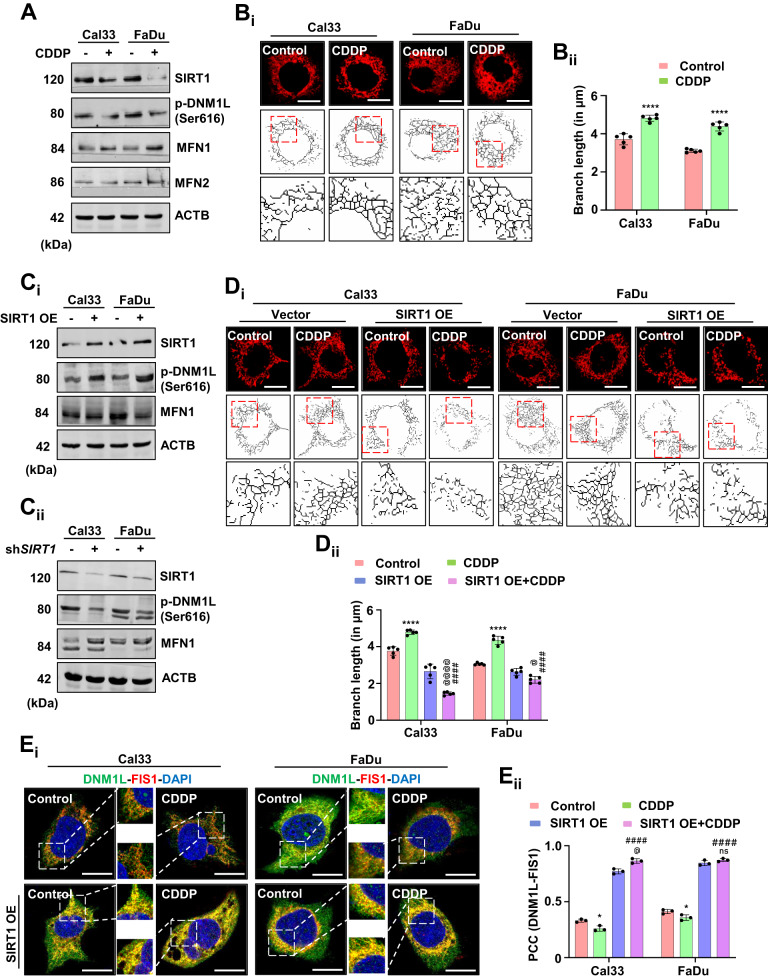
SIRT1 downregulation promotes mitochondrial hyperfusion. The expression of different protein markers, SIRT1, p-DNM1L, MFN1 and MFN2 in oral cancer cells were analyzed for confirmation of mitochondrial hyperfusion (**A**). Mitochondrial skeleton analysis was performed to determine mitochondrial branch length (**B**_**i**_ and **B**_**ii**_). The expression of SIRT1, p-DNM1L and MFN1 were analyzed in SIRT1 overexpressed (**C**_**i**_) and knockdown cells (**C**_**ii**_). Further confirmation of mitochondrial hyperfusion was done by measuring mitochondrial branch length in SIRT1 overexpressed cells (**D**_**i**_ and **D**_**ii**_). Re-induction of mitochondrial fission in the SIRT1 overexpressed cells was analyzed by studying the co-localization of DNM1L-FIS1 (**E**_**i**_ and **E**_**ii**_). Data represented in mean ± SD; students *t*-test for two variables and two-way ANOVA for multiple variables. The *p*-value > 0.05 was considered not significant (ns), *p-value < 0.05, **p-value < 0.01, ***p-value < 0.001 and ****p-value < 0.0001; ^#^*p*-value was the comparison of treatment group with the inhibitor-cotreated group with a similar level of significance. ^#,^^@^*p*-value was the comparison of the inhibitor group with the inhibitor-cotreated group with a similar significance level. The scale bar represents 10 µm.

SIRT1 plays an essential role during cellular homeostasis by maintaining mitochondrial dynamics. Hence, we investigated the involvement of SIRT1 in mitochondrial dynamics under non-apoptotic doses of CDDP. With CDDP treatment, the expression of SIRT1 and its downstream mitochondrial fission regulator DNM1L were downregulated; while mitochondrial fusion mediator MFN1 was upregulated in Cal33 and FaDu cells. Interestingly, the expression of MFN2 was found to be unaltered (Fig. 2A and Supplementary Fig. S3A). Further, the mitochondrial skeleton analysis also supported our finding as a visible increase in mitochondrial branch length was observed, indicating mitochondrial hyperfusion (Fig. 2B_i_, B_ii_). Overexpression of SIRT1 facilitated subsequent activation of DNM1L, indicating the re-introduction of mitochondrial fission to nullify mitochondrial hyperfusion (Fig. 2C_i_ and Supplementary Fig. S3B). In the sh*SIRT1* condition, decreased p-DNM1L expression and increased MFN1 expression was observed leading to a speculation that SIRT1 directly regulates the expression of mitochondrial fission and fusion factors; subsequently leading to mitochondrial hyperfusion (Fig. 2C_ii_ and Supplementary Fig. S3C). The rescue from mitochondrial hyperfusion was further confirmed from the mitochondrial skeleton analysis in the SIRT1 overexpressed cells (Fig. 2D_i_, D_ii_). Further validation of re-insisted mitochondrial fission was done by evaluating the co-localization of mitochondrial fission mediators DNM1L and FIS1 (Fig. 2E_i_, E_ii_). Altogether, the findings suggested that SIRT1 downregulation inhibits mitochondrial fission and promotes hyperfusion under non-apoptotic doses of CDDP.

### Mitochondrial hyperfusion promotes mito-bulb formation to stabilize IM cristae structure and delays the release of CYCS

**Fig. 3:**
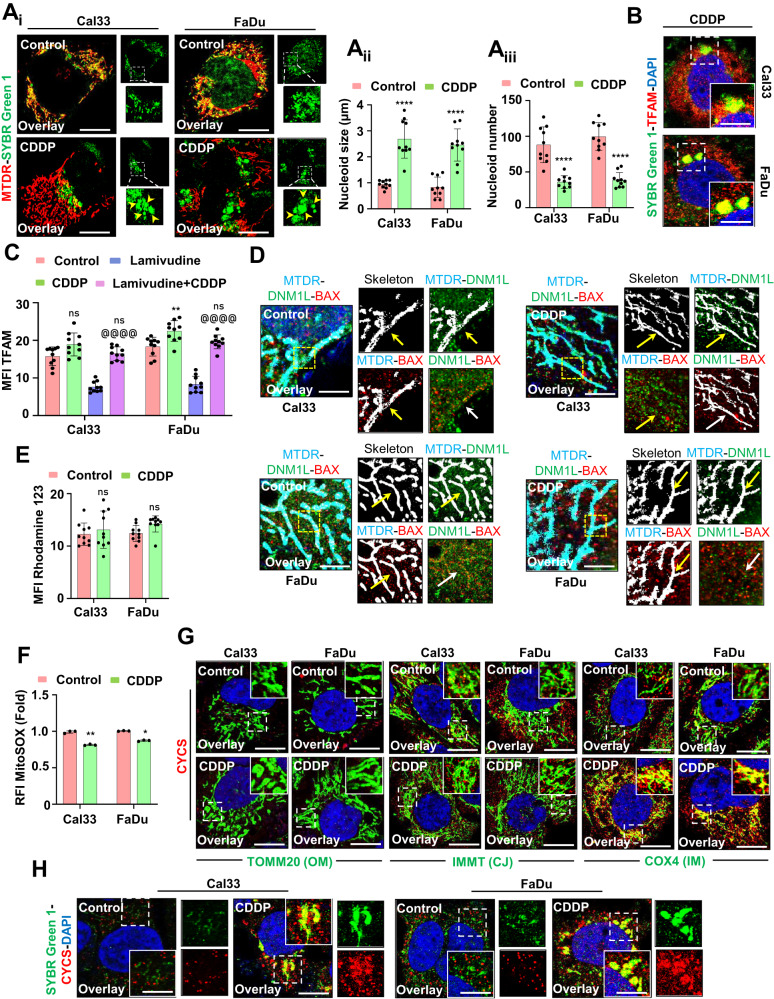
Mitochondrial hyperfusion associated mito-bulb formation entraps CYCS in the IM of mitochondria. The mitochondrial nucleoid clustering was analyzed by SYBR Green 1 staining (**A**_**i**_); the nucleoid size (**A**_**ii**_) and nucleoid number (**A**_**iii**_) were represented. The co-localization of SYBR Green 1 and TFAM was done by confocal microscopy (**B**). The expression of TFAM was analyzed in the presence of lamivudine (**C**). The DNML1-BAX complex formation was further visualized in a confocal microscope (**D**). Further investigation of mitochondrial membrane potential was done by Rhodamine 123 staining (**E**); while mitochondrial superoxide was measured by MitoSOX staining (**F**). The co-localization of CYCS in the mitochondria was visualized by colocalizing with TOMM20, IMMT and COX4 (**G**). The nucleoid entrapping of CYCS was investigated by co-localizing SYBR Green 1 with CYCS (**H**). Data represented in mean ± SD; students *t*-test for two variables and two-way ANOVA for multiple variables. The *p*-value > 0.05 was considered not significant (ns), *p-value < 0.05, **p-value < 0.01, ***p-value < 0.001 and ****p-value < 0.0001. ^@^*p*-value was the comparison of the inhibitor group with the inhibitor-cotreated group with a similar level of significance. The scale bar represents 10 µm.

To explore the physiological significance of mitochondrial hyperfusion post-CDDP treatment, we investigated mitochondrial nucleoid clustering using SYBR Green 1 staining. The CDDP-treated cells displayed a small number of extremely large clustered nucleoids within the highly interconnected mitochondrial filaments in contrast with a large number of smaller nucleoids in the control cells (Fig. 3A_i_, A_ii_, A_iii_). Furthermore, we clarified the physiological significance of these enlarged mitochondrial nucleoid clustering. We first analyzed the mtDNA positioning in the enlarged nucleoids by co-staining SYBR Green I and TFAM (Fig. 3B). Further, the mtDNA stability was checked by assessing the consequence of mtDNA replication inhibitor lamivudine. The restoration of TFAM protein expression in the CDDP-treated condition, even after lamivudine exposure, ensured that mtDNA was more stable in the enlarged nucleoids (Fig. 3C). Next, we investigated the correlation between nucleoids clustering and mitochondrial fission in the CDDP-treated cells. It has been reported that smaller dispersed mitochondrial nucleoids mark sites for mitochondrial fission. The fission site orchestrates the DNM1L-BAX complex to make mitochondrial membrane permeable (MMP) as a precursor for apoptosis. The CDDP-treated hyperfused mitochondrial network was found to be devoid of DNM1L-BAX complex formation (Fig. 3D). Trailing to the previous findings, we checked the MMP (Fig. 3E) and mitochondrial superoxide generation (Fig. 3F) by rhodamine 123 and MitoSOX staining respectively. We further characterized the mitochondrial bulb-like structures (mito-bulbs) formed after CDDP treatment. The co-staining of mitochondrial OM protein TOMM20, cristae junction protein IMMT, and IM respiratory protein COX4 with CYCS revealed that CYCS is stacked in the IM of mitochondria (as CYCS colocalized with COX4) might be due to the IM cristae stabilization in the CDDP-treated cells (Fig. 3G). Further, these enlarged mito-bulbs were found to entrap CYCS, restricting their mitochondrial release (Fig. 3H). These results suggested that mtDNA is tightly stacked in the mito-bulb like structures that trap CYCS by stabilizing IM cristae that might inhibit apoptosis when treated with CDDP.

### SIRT1 activation inhibits mito-bulb formation to facilitate mitochondrial release of CYCS

**Fig. 4:**
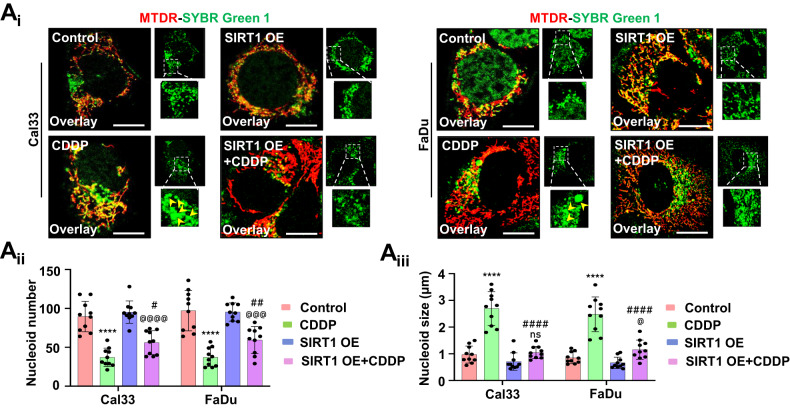
SIRT1 overexpression inhibits mito-bulb formation. The mitochondrial nucleoid clustering was analyzed by SYBR Green 1 staining (**A**_**i**_); the nucleoid number (**A**_**ii**_) and nucleoid size (**A**_**iii**_) were counted in the SIRT1 overexpressed conditions. Data represented in mean ± SD; students *t*-test for two variables and two-way ANOVA for multiple variables. The *p*-value > 0.05 was considered not significant (ns), *p-value < 0.05, **p-value < 0.01, ***p-value < 0.001 and ****p-value < 0.0001. ^#^*p*-value was the comparison of the treatment group with the inhibitor-cotreated group with a similar level of significance. ^@^*p*-value was the comparison of the inhibitor group with the inhibitor-cotreated group with a similar level of significance. The scale bar represents 10 µm.

**Fig. 5:**
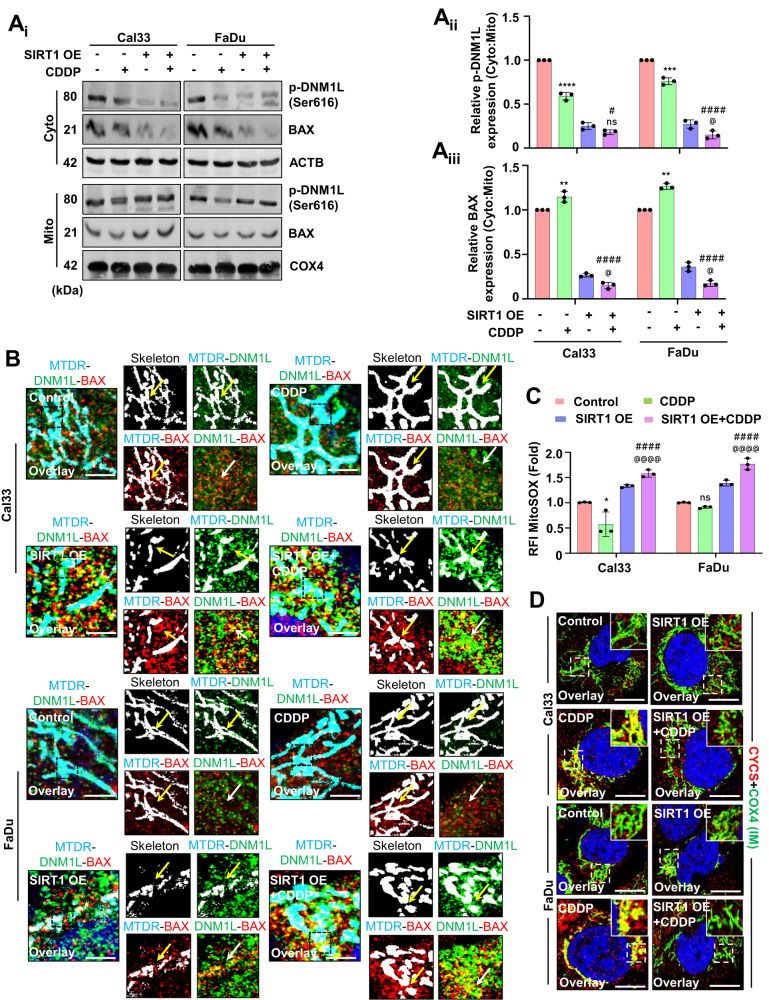
SIRT1 overexpression released CYCS by inhibiting mito-bulb formation. The subcellular fractionation was done to check the localization of p-DNM1L and BAX (**A**_**i**_–**A**_**iii**_). The DNML1-BAX complex formation was visualized under a confocal microscope in the SIRT1 overexpressed cells (**B**). The mitochondrial superoxide generation in the SIRT1 overexpression conditions was observed by MitoSOX staining (**C**). Furthermore, the CYCS entrap in the mitochondrial IM was confirmed by colocalizing it with COX4 in the SIRT1 overexpressed conditions (**D**). Data represented in mean ± SD; students *t*-test for two variables and two-way ANOVA for multiple variables. The *p*-value > 0.05 was considered not significant (ns), *p-value < 0.05, **p-value < 0.01, ***p-value < 0.001 and ****p-value < 0.0001; ^#^*p*-value was the comparison of the treatment group with the inhibitor-cotreated group with a similar level of significance. ^@^*p*-value was the comparison of the inhibitor group with the inhibitor-cotreated group with a similar significance level. The scale bar represents 10 µm.

To further specify the role of SIRT1 in the mito-bulb formation, we checked the mitochondrial nucleoids clustering in the SIRT1 overexpression conditions. The SIRT1 overexpressed cells have a large number of smaller nucleoids even after CDDP treatment (Fig. 4A_i_, A_ii_, A_iii_). As nucleoid clustering decides mitochondrial fission site and pore construction by orchestrating DNM1L-BAX complex formation, we checked the DNM1L-BAX complex formation in the SIRT1 overexpressed conditions. Subcellular fractionation was done to check the localization of p-DNM1L and BAX to the mitochondria from the cytoplasm. The SIRT1 overexpressed cells have shown enhanced p-DNM1L and BAX expression in the mitochondrial fraction indicating their mitochondrial localization (Fig. 5A_i_, A_ii_, A_iii_). The SIRT1 overexpressed cells had shown enhanced DNM1L-BAX complex formation marking site for mitochondrial fission and membrane pore formation (Fig. 5B). As the DNM1L-BAX complex was formed, we further investigated the mitochondrial superoxide generation by MitoSOX staining and found enhanced superoxide generation in the SIRT1 overexpressed cells (Fig. 5C). As the final consequence of mitochondrial nucleoids clustering trails inhibition of CYCS release by stabilization of IM cristae, we checked the co-localization of COX4_I_ with CYCS in the SIRT1 overexpressed cells. The co-localization of COX4_I_ with CYCS was reversed in the SIRT1 overexpressed cells indicating the release of CYCS from mitochondria (Fig. 5D). The findings of the above section clarified that SIRT1 overexpression facilitates the mitochondrial release of CYCS that might serve as a precursor of apoptosis.

### SIRT1-regulated mitochondrial hyperfusion is a pro-survival mechanism against cisplatin via the regulation of redox enzymes

**Fig. 6:**
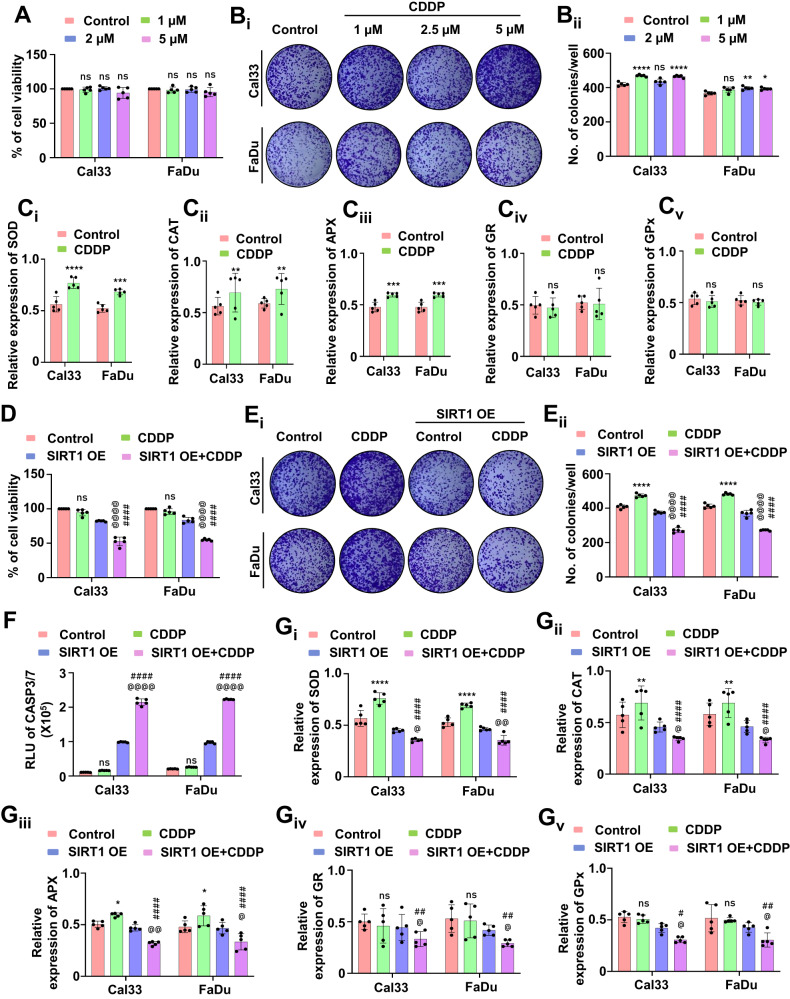
SIRT1-regulated mitochondrial hyperfusion is a pro-survival mechanism. The cell viability was assessed by MTT assay (**A**) followed by checking long-term cytotoxic potential in the clonogenic assay (**B**_**i**_ and **B**_**ii**_). Further, the antioxidant enzymatic assay was performed to check the expression of SOD (**C**_**i**_), CAT (**C**_**ii**_), APX (**C**_**iii**_), GR (**C**_**iv**_) and GP_X_ (**C**_**v**_). In SIRT1 overexpressed conditions, the cell viability was investigated by MTT assay (**D**). The clonogenic assay was performed to check the long-term cytotoxic potential when SIRT1 is overexpressed (**E**_**i**_ and **E**_**ii**_). The apoptosis induction was studied by evaluating CASP3/7 activity (**F**). The expression of antioxidant enzymes was measured by evaluating SOD (**G**_**i**_), CAT (**G**_**ii**_), APX (**G**_**iii**_), GR (**G**_**iv**_) and GP_X_ (**G**_**v**_). Data represented in mean ± SD; students *t*-test for two variables and two-way ANOVA for multiple variables. The *p*-value > 0.05 was considered not significant (ns), *p-value < 0.05, **p-value < 0.01, ***p-value < 0.001 and ****p-value < 0.0001; ^#^*p*-value was the comparison of the treatment group with the inhibitor-cotreated group with a similar level of significance. ^@^*p*-value was the comparison of the inhibitor group with the inhibitor-cotreated group with a similar level of significance.

We studied whether mitochondrial hyperfusion is a protective (pro-survival) mechanism against CDDP. Primarily, we checked the cell viability with different doses of CDDP and found no significant cell death post-treatment (Fig. 6A). The long-term cytotoxicity of the drug was further tested by clonogenic assay. No substantial reduction in the number of clones was observed, signifying the pro-survival mechanism under drug treatment (Fig. 6B_i_, B_ii_). As mitochondrial superoxide was found to play a crucial role in the regulation of apoptosis, we checked the expression of NFE2L2 (a transcription factor that maintains redox homeostasis via antioxidant enzyme regulation), which was found to be upregulated (Supplementary Fig. S4A, A_i_). Inhibition of SIRT1 by genetic approach also yielded the similar trend of result in the expression of NFE2L2 suggesting the SIRT1 mediated regulation of NFE2L2 (Supplementary Fig. S4B, B_i_). Further, we checked the enzymatic expression of SOD (Fig. 6C_i_), CAT (Fig. 6C_ii_), APX (Fig. 6C_iii_), GR (Fig. 6C_iv_) and GPx (Fig. 6C_v_), which were also upregulated. To specify the role of SIRT1, we checked the cell viability after the drug treatment in the SIRT1 overexpressed cells. The overexpressed cells were found to be more sensitive to CDDP as the cell viability declined (Fig. 6D). Similarly, the clonogenic assay demonstrated a reduced number of clones in the SIRT1 overexpressed cells treated with CDDP (Fig. 6E_i_, E_ii_). The apoptosis induction was confirmed in the overexpressed cells by checking the CASP3/7 activity (Fig. 6F). Further, we checked the expression of NFE2L2, which was found to be downregulated in the SIRT1 overexpressed cells treated with CDDP (Supplementary Fig. S4C,C_i_ and D,D_i_). A similar trend of results were observed in the enzymatic expression of SOD (Fig. 6G_i_), CAT (Fig. 6G_ii_), APX (Fig. 6G_iii_), GR (Fig. 6G_iv_) and GPx (Fig. 6G_v_), confirming that SIRT1 overexpression abrogates mitochondrial hyperfusion associated apoptosis inhibition by regulating NFE2L2-antioxidant pathways.

### Low-dose priming of a novel SIRT1 activator, gallic acid, drives oral cancer cells to apoptosis via inhibiting mitochondrial hyperfusion

**Fig. 7:**
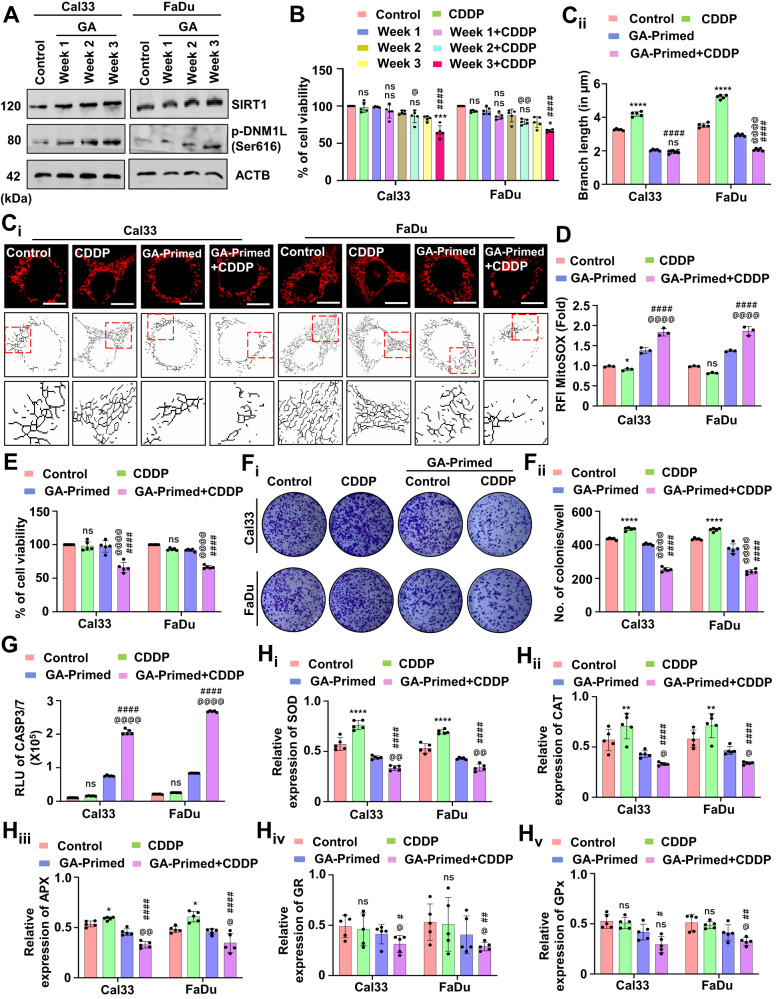
Low-dose priming of gallic acid, a SIRT1 activator, inhibits mitochondrial hyperfusion and induces apoptosis. The expression of SIRT1 and p-DNM1L was evaluated by western blotting in the GA-primed cells (**A**). The cell viability of the GA-primed cells was assessed by MTT assay (**B**). The mitochondrial hyperfusion was evaluated by evaluating mitochondrial branch length (**C**_**i**_ and **C**_**ii**_). Further evaluation of mitochondrial superoxide generation was assessed by MitoSOX staining (**D**). The cytotoxicity and long-term cytotoxicity of the GA-primed cells were evaluated by MTT (**E**) and clonogenic assay, respectively (**F**_**i**_ and **F**_**ii**_). The CASP3/7 activity (**G**) was evaluated. The expression of antioxidant enzymes was measured by evaluating SOD (**H**_**i**_), CAT (**H**_**ii**_), APX (**H**_**iii**_), GR (**H**_**iv**_) and GP_X_ (**H**_**v**_). Data represented in mean ± SD; students *t*-test for two variables and two-way ANOVA for multiple variables. The *p*-value > 0.05 was considered not significant (ns), *p-value < 0.05, **p-value < 0.01, ***p-value < 0.001 and ****p-value < 0.0001; ^#^*p*-value was the comparison of the treatment group with the inhibitor-cotreated group with a similar level of significance. ^@^*p*-value was the comparison of the inhibitor group with the inhibitor-cotreated group with a similar level of significance. The scale bar represents 10 µm.

Our virtual screening methods identified GA, a bioactive polyphenol that activates SIRT1 (Supplementary Fig. S5A and Table 1). Hence, we primed the oral cancer cells with low doses of GA for 3 weeks and checked the SIRT1 activity. Interestingly, the GA-primed cells showed higher expression of SIRT1 as well as DNM1L (Fig. 7A and Supplementary Fig. S5B). The cell viability assay demonstrated that GA-primed (not resistance to GA) cells were sensitized to cell death when treated with CDDP (Fig. 7B). Further, the mitochondrial skeleton analysis confirmed that GA-primed cells have shorter mitochondrial branch length due to reduced mitochondrial hyperfusion (Fig. 7C_i_, C_ii_). Moreover, the CDDP treated GA-primed cells also have enhanced expression of p-DNM1L suggesting the onset of mitochondrial fission (Supplementary Fig. S6A, A_i_). The mitochondrial superoxide level was also found to be upregulated in the CDDP-treated GA-primed cells as compared to only GA-primed cells (Fig. 7D). To investigate the mechanism behind such peculiar upsurge in the mitochondrial superoxide level in the CDDP-treated GA-primed cells, we checked the expression of NFE2L2. Interestingly, we found a reduced NFE2L2 expression in the CDDP-treated GA-primed cells (Supplementary Fig. S6A, A_i_) that could be the possible cause for enhancement in the mitochondrial superoxide to induce cell death. The cell viability (Fig. 7E) and clonogenic assay (Fig. 7F_i_, F_ii_) demonstrated that GA-primed cells are more sensitive towards CDDP. The apoptosis induction was checked by evaluating CASP3/7 activity (Fig. 7G). As antioxidant plays an essential role in apoptosis induction, we studied the expression of SOD (Fig. 7H_i_), CAT (Fig. 7H_ii_), APX (Fig. 7H_iii_), GR (Fig. 7H_iv_) and GPx (Fig. 7H_v_). In the GA-primed cells, when treated with CDDP, the antioxidant enzymes were found to be reduced.

**Table 1:** Identification of Gallic acid as a potent SIRT1 activator.

Name of the compound	Water Solubility			Pharmacokinetics							Drug likeliness		Medicinal chemistry
	ESOL	Ali	SILICOS-IT	GI-absorption	P-gp substrate	CYP1A2 inhibitor	CYP2C19 inhibitor	CYP2C9 inhibitor	CYP2D6 inhibitor	CYP3A4 inhibitor	Lipinski	Bio-availability score	Synthetic accessibility
Resveratrol	−3.62 (S)	−4.07 (MS)	−3.29 (S)	High	No	Yes	No	Yes	No	Yes	Yes	0.55	2.02
Curcumin	−3.94 (S)	−4.83 (MS)	−4.45 (MS)	High	No	No	No	Yes	No	Yes	Yes	0.55	2.97
Quercetin	−3.16 (S)	−3.91 (S)	−3.24 (S)	High	No	Yes	No	No	Yes	Yes	Yes	0.55	3.23
Berberine	−4.55 (MS)	−4.16 (MS)	−5.92 (MS)	High	Yes	Yes	No	No	Yes	Yes	Yes	0.55	3.14
Fisetin	−3.35 (S)	−3.35 (S)	−3.82 (S)	High	No	Yes	No	No	Yes	Yes	Yes	0.55	3.16
Apigenin	−3.94 (S)	−4.59 (MS)	−4.40 (MS)	High	No	Yes	No	No	Yes	Yes	Yes	0.55	2.96
Cianidanol	−2.22 (S)	−2.24 (S)	−2.14 (S)	High	Yes	No	No	No	No	Yes	Yes	0.55	3.5
**Gallic acid**	**−1.64 (VS)**	**−2.34 (S)**	**−0.04 (VS)**	**High**	**No**	**No**	**No**	**No**	**No**	**Yes**	**Yes**	**0.56**	**1.22**
Epicatechin	−2.22 (S)	−2.24 (S)	−2.14 (S)	High	Yes	No	No	No	No	No	Yes	0.55	3.5
Genistein	−3.72 (S)	−4.23 (MS)	−4.40 (MS)	High	No	Yes	No	No	Yes	Yes	Yes	0.55	2.87
Kaempferol	−3.31 (S)	−3.86 (S)	−3.82 (S)	High	No	Yes	No	No	Yes	Yes	Yes	0.55	3.14
Ellagic acid	−2.94 (S)	−3.66 (S)	−3.35 (S)	High	No	Yes	No	No	No	No	Yes	0.55	3.17
Caffeic acid	−1.89 (VS)	−2.38 (S)	−0.71 (VS)	High	No	No	No	No	No	No	Yes	0.56	1.81
Luteolin	−3.71 (S)	−4.51 (MS)	−3.82 (S)	High	No	Yes	No	No	Yes	Yes	Yes	0.55	3.02

The bold values represent the data for Gallic acid, which is the identified compound of this study.

**Fig. 8:**
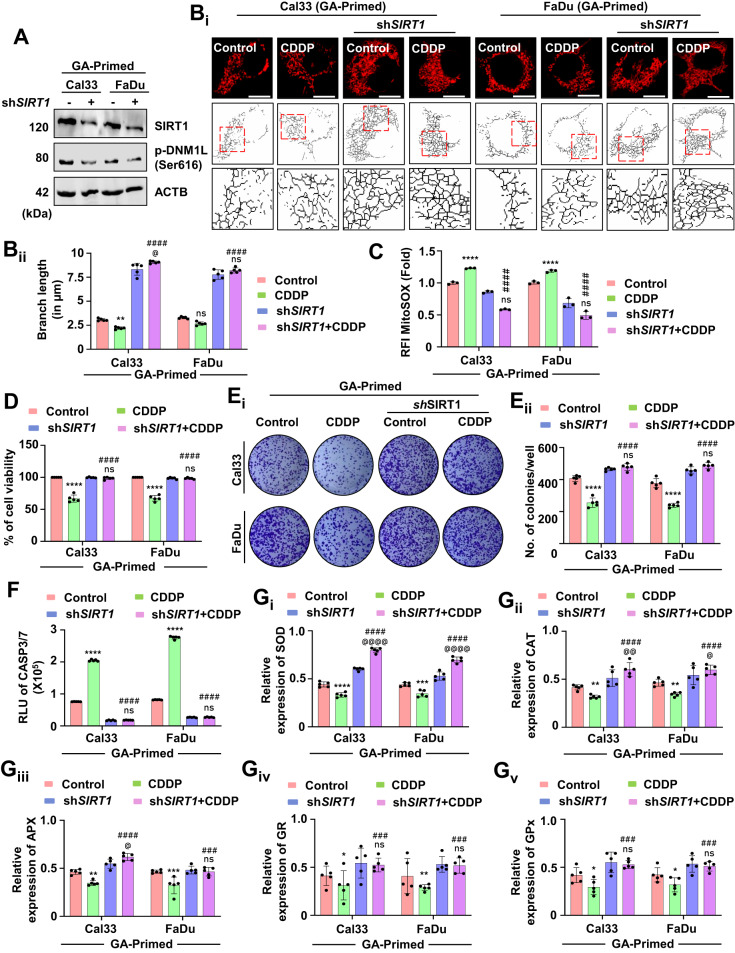
SIRT1 knockdown in the GA-primed cells rescue the cells from apoptosis by re-inducing mitochondrial hyperfusion. To confirm the role of SIRT1, a knockdown of SIRT1 in the GA-primed cells was done. Firstly, the expression of SIRT1 and p-DNM1L was evaluated (**A**). The mitochondrial branch length was calculated by skeletonizing the images to check mitochondrial hyperfusion (**B**_**i**_ and **B**_**ii**_). Further, the MitoSOX staining quantified mitochondrial superoxide generation in the SIRT1 knockdown cells (**C**). The cell viability (**D**) and clonogenic assay (**E**_**i**_ and **E**_**ii**_) were performed in the SIRT1 knockdown cells. The apoptosis induction potential was evaluated by CASP3/7 Glo assay (**F**). The expression of antioxidant enzymes was measured by evaluating SOD (**G**_**i**_), CAT (**G**_**ii**_), APX (**G**_**iii**_), GR (**G**_**iv**_) and GP_X_ (**G**_**v**_). Data represented in mean ± SD; students *t*-test for two variables and two-way ANOVA for multiple variables. The *p*-value > 0.05 was considered not significant (ns), *p-value < 0.05, **p-value < 0.01, ***p-value < 0.001 and ****p-value < 0.0001; ^#^*p*-value was the comparison of treatment group with the inhibitor-cotreated group with a similar level of significance. ^@^*p*-value was the comparison of the inhibitor group with the inhibitor-cotreated group with a similar level of significance. The scale bar represents 10 µm.

Next, in the GA-primed cells, the knockdown of SIRT1 abrogated the expression of DNM1L (Fig. 8A and Supplementary Fig. S6B). SIRT1 knockdown also reintroduced the mitochondrial hyperfusion in the GA-primed cells as evidenced by increasing branch length (Fig. 8B_i_, B_ii_). The mitochondrial superoxide level was also decreased in the SIRT1 knockdown GA-primed cells (Fig. 8C). An enhanced cell viability (Fig. 8D) and higher number of colonies (Fig. 8E_i_, E_ii_) specified the role of SIRT1 in the GA-primed cells. The reversal of apoptosis was evaluated by checking CASP3/7 activity (Fig. 8F). Further, the expression of antioxidant enzymes; SOD (Fig. 8G_i_), CAT (Fig. 8G_ii_), APX (Fig. 8G_iii_), GR (Fig. 8G_iv_) and GPx (Fig. 8G_v_) were also reversed in the GA-primed cells when SIRT1 was knockdown. The above findings suggested that low-dose priming with SIRT1 activator GA inhibits mitochondrial hyperfusion for induction of apoptosis, and the process was rescued under genetic ablation of SIRT1 in the GA-primed cells.

### SIRT1 activation inhibits mitochondrial hyperfusion and re-sensitizes the cisplatin-resistant oral cancer PGCCs to apoptosis

**Fig. 9:**
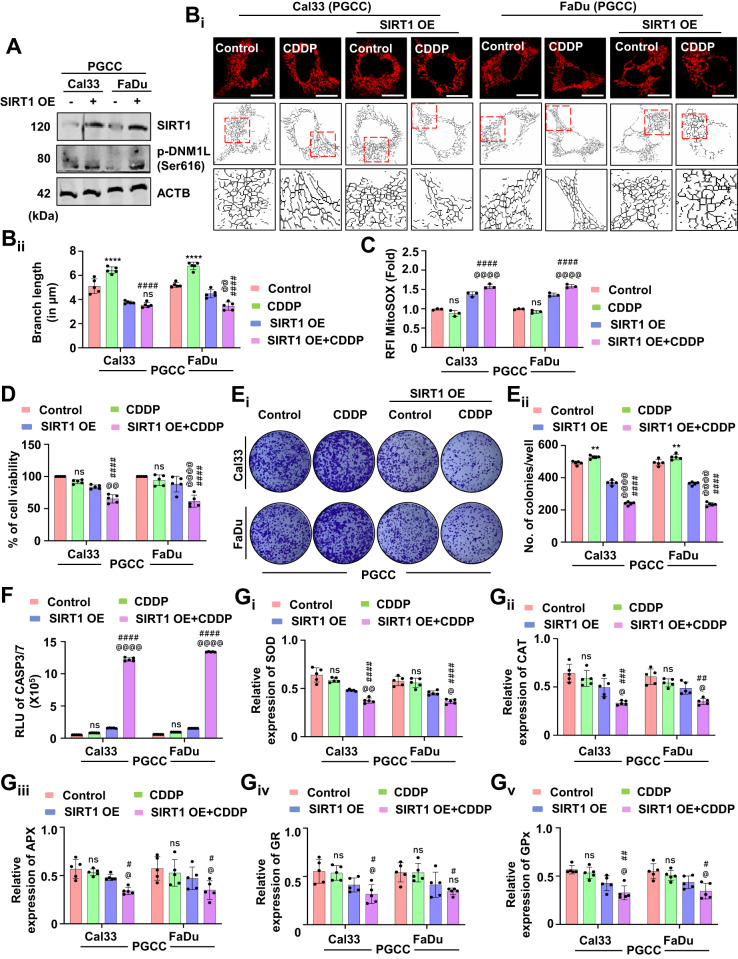
SIRT1 overexpression induces apoptosis in oral cancer-derived PGCCs by inhibiting mitochondrial hyperfusion. The expression of SIRT1 and p-DNM1L was evaluated by western blotting in oral cancer-derived PGCCs (**A**). The mitochondrial skeleton analysis exhibited the mitochondrial branch length confirming mitochondrial hyperfusion (**B**_**i**_ and **B**_**ii**_). The generation of mitochondrial superoxide in the PGCCs was evaluated by MitoSOX staining (**C**). The cytotoxicity and long-term cytotoxicity of the PGCCs were evaluated by MTT (**D**) and clonogenic assay, respectively (**E**_**i**_ and **E**_**ii**_). The apoptosis induction potential was evaluated by CASP3/7 Glo assay (**F**). The expression of antioxidant enzymes was measured by evaluating SOD (**G**_**i**_), CAT (**G**_**ii**_), APX (**G**_**iii**_), GR (**G**_**iv**_) and GP_X_ (**G**_**v**_) in oral cancer-derived PGCCs. Data represented in mean ± SD; students *t*-test for two variables and two-way ANOVA for multiple variables. The *p*-value > 0.05 was considered not significant (ns), *p-value < 0.05, **p-value < 0.01, ***p-value < 0.001 and ****p-value < 0.0001; ^#^*p*-value was the comparison of the treatment group with the inhibitor-cotreated group with a similar level of significance. ^@^*p*-value was the comparison of the inhibitor group with the inhibitor-cotreated group with a similar level of significance. The scale bar represents 10 µm.

As mitochondrial hyperfusion acts as a pro-survival mechanism against drug-induced apoptosis, we were keen to examine the role of mitochondrial hyperfusion in CDDP resistant oral cancer cells. In our initial observation, we found reduced SIRT1 expression in the oral cancer-derived PGCCs (Fig. 1E). Hence, we overexpressed SIRT1 in the PGCCs and found subsequent activation of DNM1L (Fig. 9A and Supplementary Fig. S7A). Further, the skeleton analysis demonstrated reduced mitochondrial branch length in the SIRT1 overexpressed PGCCs (Fig. 9B_i_, B_ii_), suggesting inhibition of mitochondrial hyperfusion. The mitochondrial superoxide level was found to be increased in the SIRT1 overexpressed PGCCs (Fig. 9C). The cell viability (Fig. 9D) and the number of clones (Fig. 9E_i_, E_ii_) were also found to be reduced in the SIRT1 overexpressed PGCCs. Enhanced CASP3/7 activity also signified induction of apoptosis in the PGCCs post SIRT1 overexpression (Fig. 9F). Reduced expression of antioxidant enzymes; SOD (Fig. 9G_i_), CAT (Fig. 9G_ii_), APX (Fig. 9G_iii_), GR (Fig. 9G_iv_) and GPx (Fig. 9G_v_) were also observed in the SIRT1 overexpressed PGCCs. The above findings suggested the critical involvement of SIRT1-regulated mitochondrial hyperfusion to re-sensitize oral cancer-derived PGCCs to CDDP-induced apoptosis.

### Synergistic treatment of gallic acid with cisplatin inhibits mitochondrial hyperfusion for the onset of apoptosis in oral cancer PGCCs

**Fig. 10:**
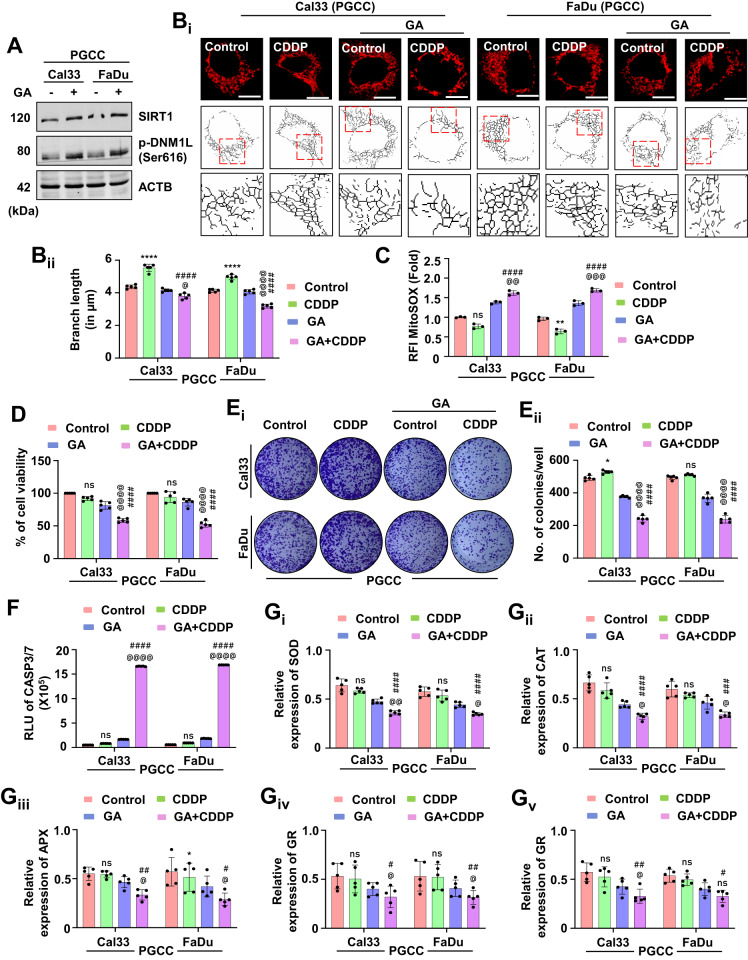
Synergistic treatment of GA with CDDP re-sensitizes oral cancer-derived PGCCs to apoptosis via inhibition of mitochondrial hyperfusion. The synergistic effect of GA in the CDDP resistance PGCCs was evaluated by checking the expression of SIRT1 and p-DNM1L (**A**). Confirmation of inhibition of mitochondrial hyperfusion was evaluated by mitochondrial skeleton analysis (**B**_**i**_ and **B**_**ii**_). The MitoSOX staining confirmed the generation of mitochondrial superoxide (**C**). The MTT assay (**D**) confirmed inhibition of cell viability in the co-treated groups. Further confirmation of cytotoxicity was done via clonogenic assay (**E**_**i**_ and **E**_**ii**_). The re-sensitization to apoptosis was done by calculating the CASP3/7 activity (**F**). The expression of antioxidant enzymes was measured by evaluating SOD (**G**_**i**_), CAT (**G**_**ii**_), APX (**G**_**iii**_), GR (**G**_**iv**_) and GP_X_ (**G**_**v**_). Data represented in mean ± SD; students *t*-test for two variables and two-way ANOVA for multiple variables. The *p*-value > 0.05 was considered not significant (ns), *p-value < 0.05, **p-value < 0.01, ***p-value < 0.001 and ****p-value < 0.0001; ^#^*p*-value was the comparison of the treatment group with the inhibitor-cotreated group with a similar level of significance. ^@^*p*-value was the comparison of the inhibitor group with the inhibitor-cotreated group with a similar level of significance. The scale bar represents 10 µm.

SIRT1 overexpression re-sensitizes oral cancer-derived PGCCs to CDDP-induced apoptosis. Here, we studied whether GA can also re-sensitize the PGCCs to apoptosis. Firstly, we checked the SIRT1 and DNM1L activation potential of GA in the PGCCs (Fig. 10A and Supplementary Fig. S8A). The skeleton analysis suggested inhibition of mitochondrial hyperfusion in the GA synergized PGCCs even after treatment of CDDP (Fig. 10B_i_, B_ii_). The GA synergized PGCCs also exhibited enhanced mitochondrial superoxide generation (Fig. 10C). Moreover, reduced cell viability (Fig. 10D) along with the lesser number of clones (Fig. 10E_i_, E_ii_) suggested re-sensitization of PGCCs to apoptosis after synergistic treatment of GA and CDDP. An enhanced CASP3/7 activity indicated induction of apoptosis was also observed in the PGCCs (Fig. 10F). in the GA-synergized conditions, lower expression of antioxidant enzymes; SOD (Fig. 10G_i_), CAT (Fig. 10G_ii_), APX (Fig. 10G_iii_), GR (Fig. 10G_iv_) and GPx (Fig. 10G_v_) were observed. The observations of our findings suggested that synergistic treatment of GA with CDDP inhibits mitochondrial hyperfusion for the subsequent induction of apoptosis by modulating the antioxidant system.

## Discussion

SIRT1 functions as a dual regulator of survival and cell death induction in cancer cells [15, 16]. In the tumor and cancer-specific context, SIRT1 activation or inhibition provides a better therapeutic approach with critical insights into understanding the molecular involvement and aspects of SIRT1 regulating small-molecular drug candidates [17]. In the present study, we have focused on unrevealing the intricate role of SIRT1 as a tumor suppressor gene in oral cancer during chemotherapy and the molecular aspects that SIRT1 regulates during therapeutic intervention with special insight into mitochondrial dynamics. The present study showed that SIRT1, a tumor suppressor gene, is downregulated in oral cancer during the therapeutic intervention and facilitates drug resistance [16].

What physiological role does mitochondrial dynamics play during cellular homeostasis has long been considered a critical question that needs extensive clarification. During chemotherapeutic stress, mitochondrial dynamics is found to be altered for the maintenance of cellular homeostasis [8, 25, 26]. Although mitochondrial fission has already been reported as a stress response mechanism, seldom facts about mitochondrial hyperfusion as a stress response mechanism has been discussed [8]. Mitochondrial hyperfusion enforces optimal ATP production through OXPHOS under stimuli for subsequent cell survival [8, 27]. Our study showed that downregulation of SIRT1 during the treatment of sub-lethal doses of CDDP exhibit mitochondrial hyperfusion by inhibiting DNM1L-mediated mitochondrial fission and is associated with drug resistance and cell viability. Further, the mechanistic insight into the physiological significance of mitochondrial hyperfusion has revealed the dynamic distribution and clustering of mitochondrial nucleoids mimicking mito-bulb like structures with tightly packed cristae to entrap CYCS in the CDDP-treated cells [28, 29]. We also clarified previous reports that mitochondrial fission prevents mitochondrial nucleoid clustering as SIRT1 overexpression rescues the nucleoid clustering phenomenon.

Another question about nucleoid clustering leading to mito-bulb formation with tightly packed cristae needs further clarification. It has previously been reported that mtDNA directs mito-bulb formation with stacked cristae reformation. mtDNA is believed to be closely associated with mitochondrial IM; hence nucleoid clusters mimicking mito-bulb formation are closely associated with IM [30, 31]. Interestingly, our observation signified that SIRT1 overexpression inhibits nucleoid clustering and mito-bulb formation as a result of the inhibition of mitochondrial fission. It is also believed that mito-bulb formation might affect mitochondrial ATPase oligomerization as it has a regulatory function on mitochondrial cristae reformation [32]. Recent reports have opined that tightly regulated cristae in hyperfused mitochondria protect it against autophagic degradation and apoptotic cell death during external stimuli and adverse cellular conditions [33–35]. However, the physiological role of these cristae rearrangements with mito-bulb formation remains to be convincingly examined.

Mitochondrial fission is vital for the induction of apoptosis as it directs DNM1L to induce constrictions on the mitochondrial membrane. DNM1L directly affects the membrane integrity of mitochondrial OM [2]. Further, DNM1L tips BAX to the constriction sites for the induction of mitochondrial membrane pores to facilitate the mitochondrial release of CYCS for the subsequent induction of apoptosis. DNM1L-mediated cristae reformation is responsible for the release of CYCS during apoptosis [36–38]. CYCS are known to be stacked in the IM cristae, which are expected to be released post-induction of apoptosis [28, 29]. The topological distribution of these mitochondrial structures distinctively regulate BAX-mediated CYCS release [38]. SIRT1 inhibition directs the submitochondrial distribution of enlarged mito-bulbs that entrap CYCS to hinder apoptosis. As the DNM1L-BAX complex is indispensable for apoptosis induction, our study found that SIRT1 overexpression enables DNM1L-BAX mediated loss of mitochondrial membrane permeability and mitochondrial superoxide generation as a precursor of apoptosis induction with a remarkable notion to NFE2L2 downregulation. Our findings also suggested that SIRT1 directly regulates the mitochondrial hyperfusion and antioxidant enzyme expression in a MFN1 and NFE2L2 dependent manner respectively. Interestingly, the enzymatic expression of SOD, CAT, APX, GR and GP_X_ were also found to be downregulated, signifying their regulation by NFE2L2 [24, 39]. While most of the observed reports have demonstrated that SIRT1 upregulates the antioxidant enzymes, we observed a contrasting result. Supporting data suggested that GA-mediated SIRT1 activation might be directing the DNM1L and FIS1 to the peripheral constriction site on the mitochondria leading to asymmetric fission rather than symmetric mitochondrial fission to activate mitophagy (occurrence of mitochondrial biogenesis is limited). Based on these facts, we have reported downregulation of NFE2L2 with activation of SIRT1.

**Fig. 11:**
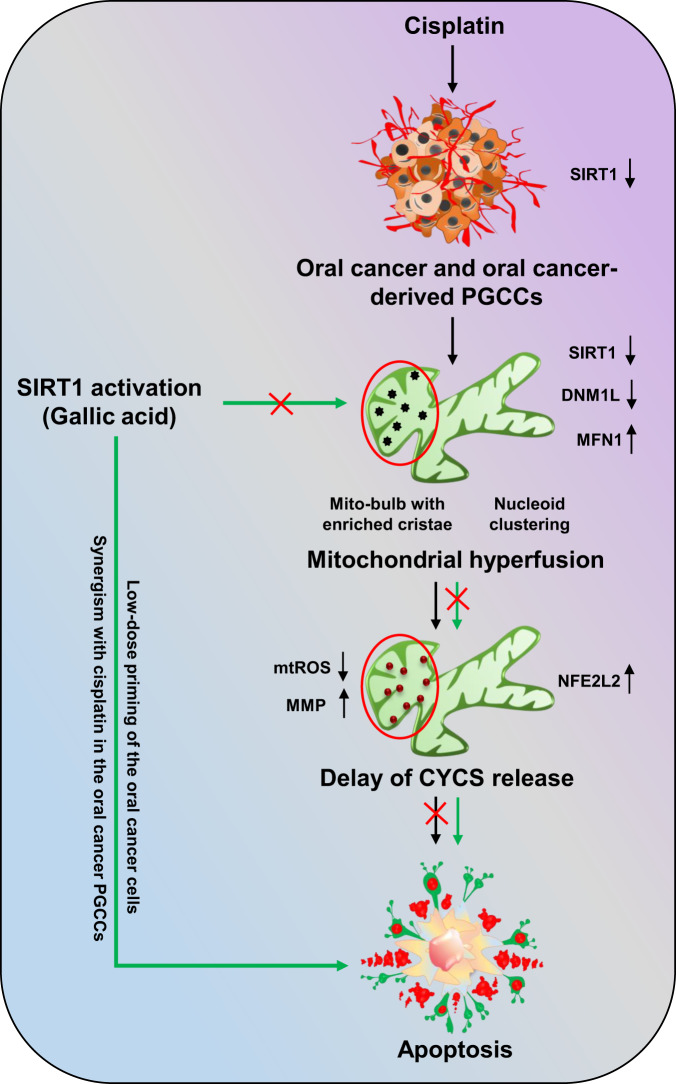
Overview of the present work. Non-apoptotic doses of CDDP downregulate the expression of SIRT1 in oral cancer and CDDP-resistance oral cancer-derived PGCCs. Downregulation of SIRT1 promotes mitochondrial hyperfusion by inhibition of DNML1-mediated fission events. The hyperfused mitochondrial network possesses mito-bulb like structures that stabilise IM cristae and entrap CYCS to inhibit apoptosis. SIRT1 activation rescues the cells for mitochondrial hyperfusion and induces apoptosis. Moreover, SIRT1 activation also re-sensitize CDDP resistance PGCCs to apoptosis by reintroducing mitochondrial fission.

Further confirmation of SIRT1-regulated apoptosis induction in oral cancer cells was confirmed in GA-primed cells in basal as well as SIRT1 knockdown conditions. Moreover, crucial role of SIRT1 activation in sensitizing the cancer cells to apoptosis via inhibition of mitochondrial hyperfusion was deciphered in CDDP-resistant oral cancer cells. On a brief note, our study revealed the critical involvement of SIRT1 in regulating mitochondrial hyperfusion, mtDNA distribution and mitochondrial cristae reformation, which physiologically energies induction of apoptosis (Fig. 11). However, further investigation is warranted to delineate the mechanistic involvement of SIRT1 as a directive target for mitochondrial dynamics with the commendable therapeutic intervention against oral cancer.

### Supplementary information

Supplementary Data

Original Data File

aj-checklist

## Data Availability

The original contributions presented in the study are included in the article; further inquiries can be directed to the corresponding author.
