# Supplementary material for: SIRT1 inhibits mitochondrial hyperfusion associated mito-bulb formation to sensitize oral cancer cells for apoptosis in a mtROS-dependent signalling pathway
Source: Cell Death Dis. 2023 Nov 10;14(11):732. doi: 10.1038/s41419-023-06232-x (PMC10638388; doi:10.1038/s41419-023-06232-x)
Supplement: Supplementary file 1 — Supplementary Data [file 41419_2023_6232_MOESM1_ESM.docx]

**Supplementary Figure 1 (S1)**


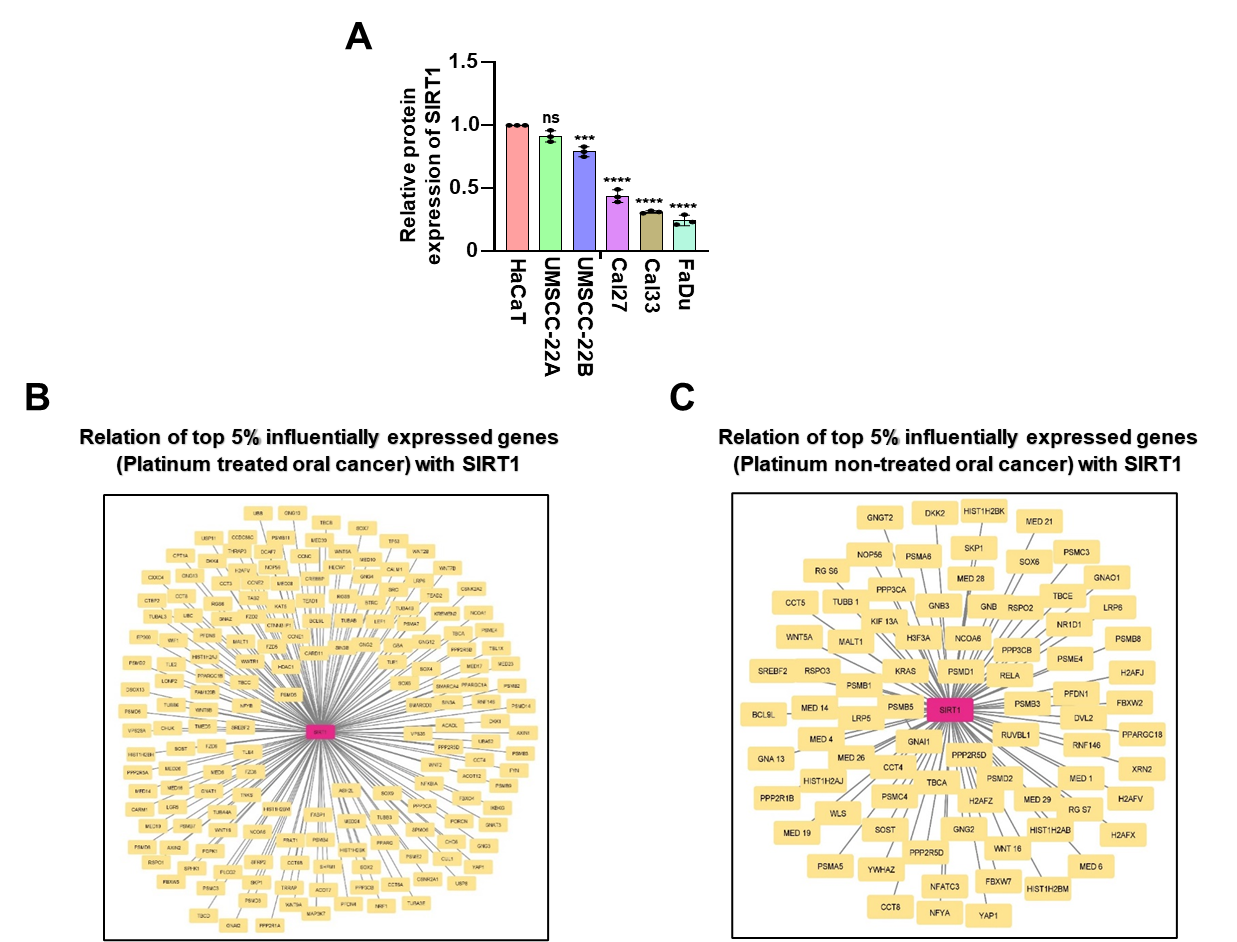


**Supplementary Fig. S1.** **Interaction of SIRT1 with influential genes in platinum-treated and platinum non-treated oral tumors**

The quantification of western blot was represented (S1A). The cytoscape was used to check the interation of SIRT1 with the top 5% influential genes in platinum-treated (S1B) and platimun non-treated (S1C) oral tumors. Schomberg’s chemo-informatics analysis was used to identify the influential genes.

**Supplementary Figure 2 (S2)**


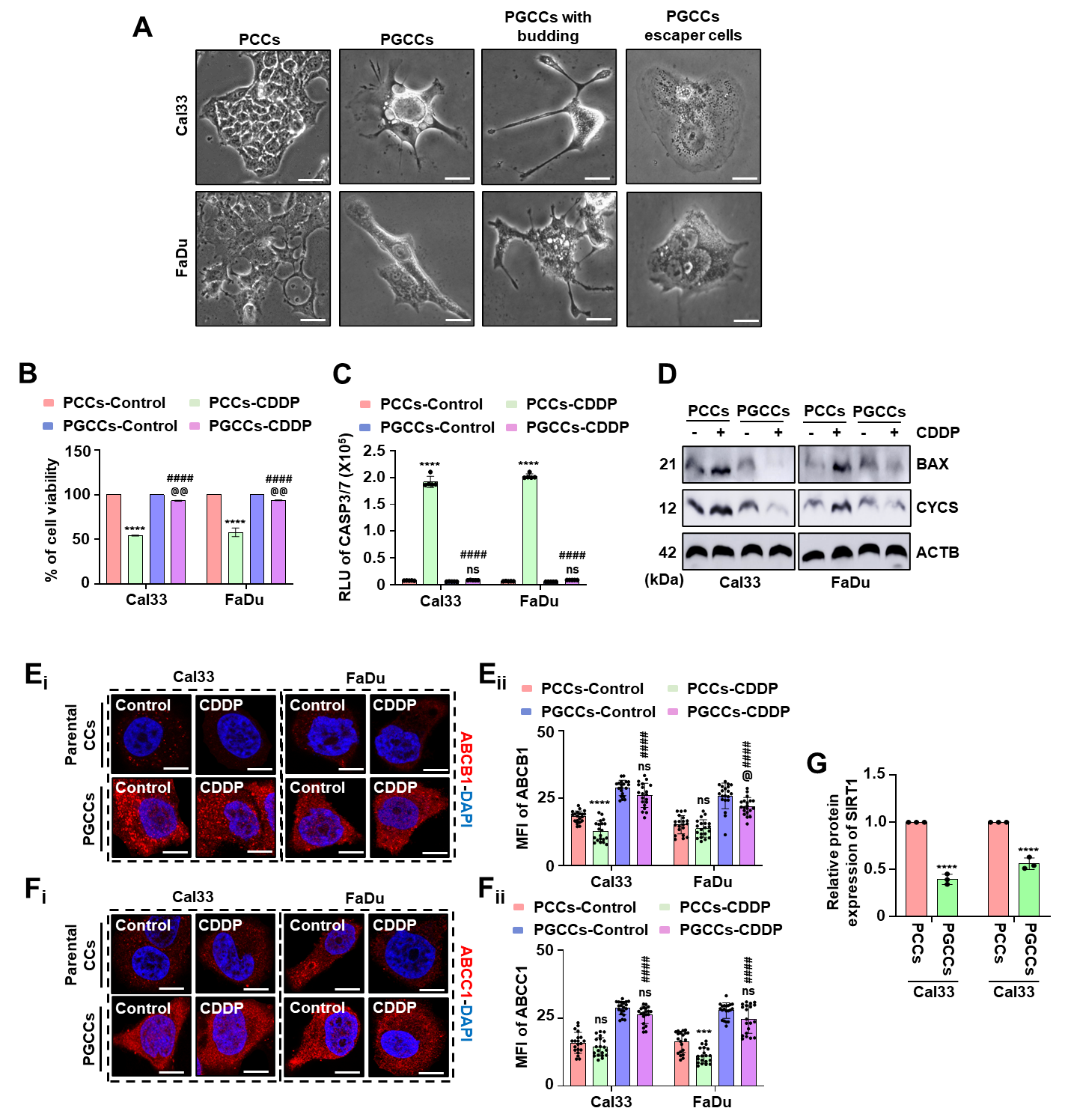


**Supplementary Fig. S2.** **Characterization and cisplatin resistance of PGCCs**

Primarily, we checked the morphological characteristics of PGCCs. Enlarged cells with multinucleated structures were observed during the recovery phase post treatment of CDDP (S2A). The MTT assay was performed to check the cell viability of the PGCCs upon treatment of CDDP (S2B). Further clarification of apoptosis inhibition was done by checking the CASP3/7 activity (S2C) along with expression analysis of BAX and CYCS by western blotting (S2D). Further confirmation of drug resistance was measured by checking the expression of ABCB1 (S2E_i_ and E_ii_) and ABCC1 (S2F_i_ and F_ii_). The quantification of western blot was represented (S2G). Data represented in mean ± SD; two-way ANOVA for multiple variables. The p-value > 0.05 was considered not significant (ns), ***p-value < 0.001 and ****p-value < 0.0001; ^#^p-value was the comparison of the treatment group with the inhibitor-cotreated group with a similar level of significance. ^@^p-value was the comparison of the inhibitor group with the inhibitor-cotreated group with a similar level of significance. The scale bar represents 50 µm (S2A) and 10 µm (S2E_i_ and F_i_).

**Supplementary Figure 3 (S3)**

**
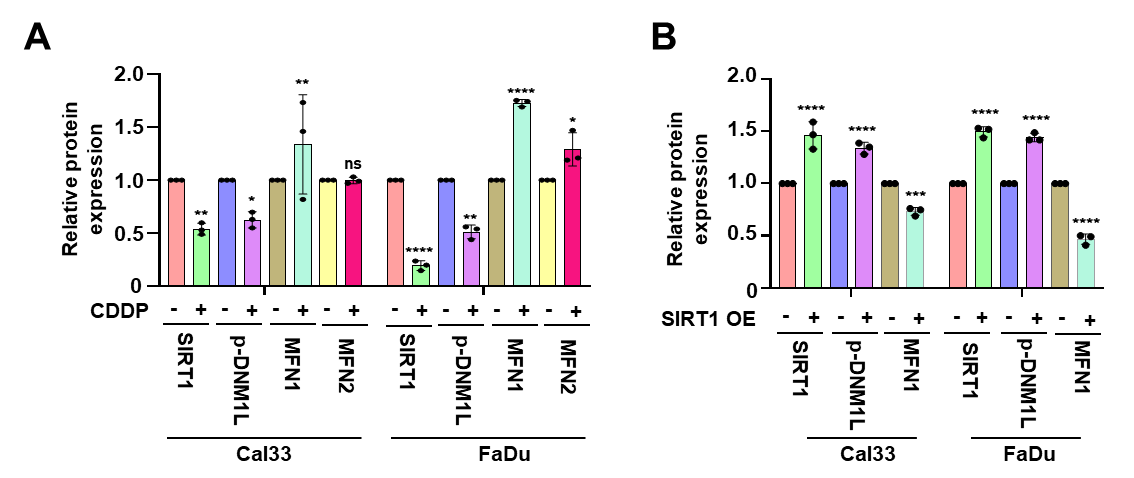
**

**Supplementary Fig. S3.** The quantification of western blots such as SIRT1, p-DNM1L, MFN1 and MFN2 in the presence of CDDP was represented (S3A). Further the quantification of western blots such as SIRT1, p-DNM1L and MFN1 was represented (S3B).

**
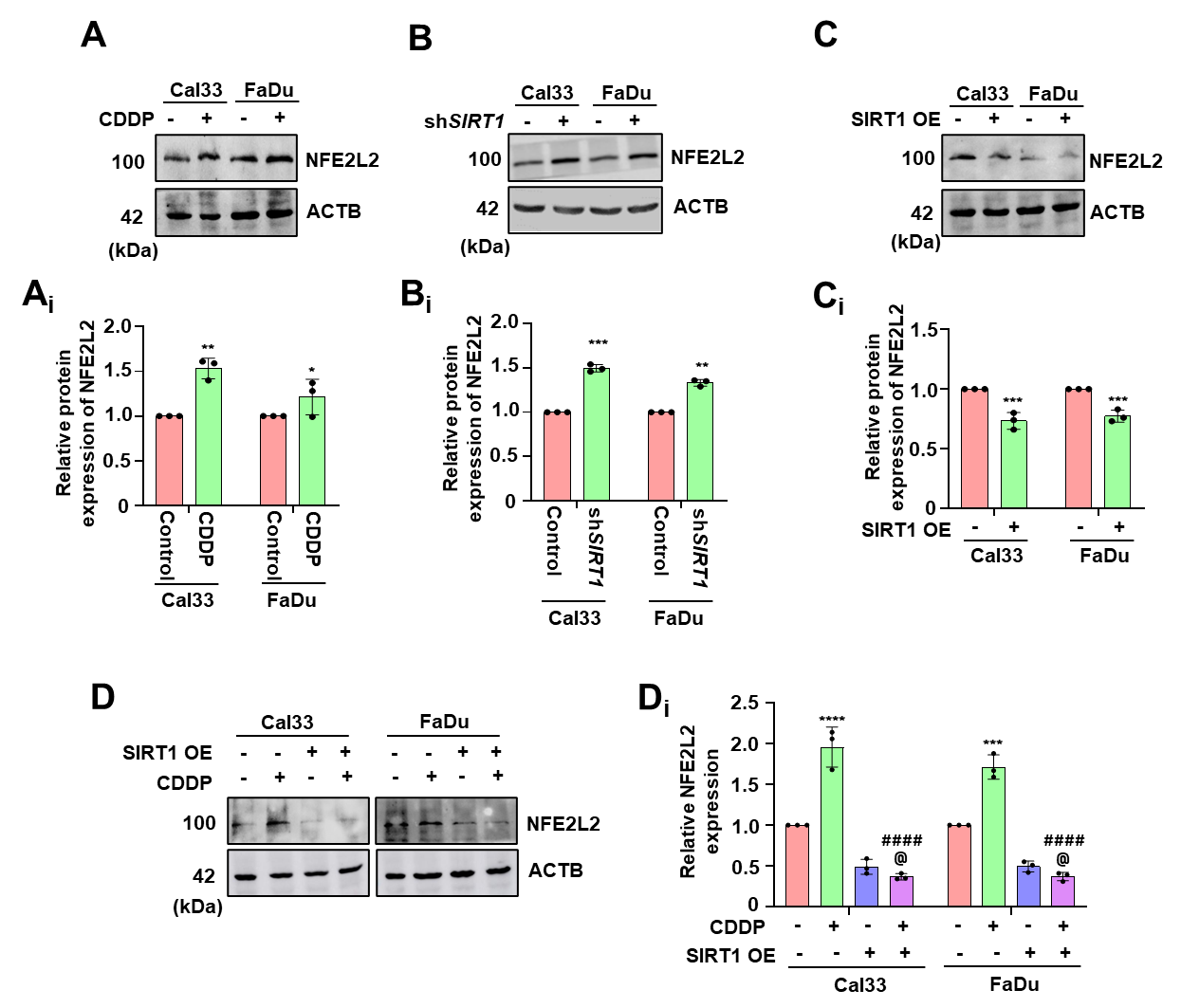
Supplementary Figure 4 (S4)**

**Supplementary Fig. S4.** The protein expression of NFE2L2 was evaluated by western blotting. The NFE2L2 expression was analyzed in the presence of CDDP and found to be upregulated (S4A and A_i_). The similar results were found in the SIRT1 knockdown conditions (S4B and B_i_). The expression of NFE2L2 was found to be downregulated in the SIRT1 overexpressed conditions (S4C and C_i_). The Expression of NFE2L2 in the CDDP-treated SIRT1 OE cells (S4D and D_i_).

**Supplementary Figure 5 (S5)**


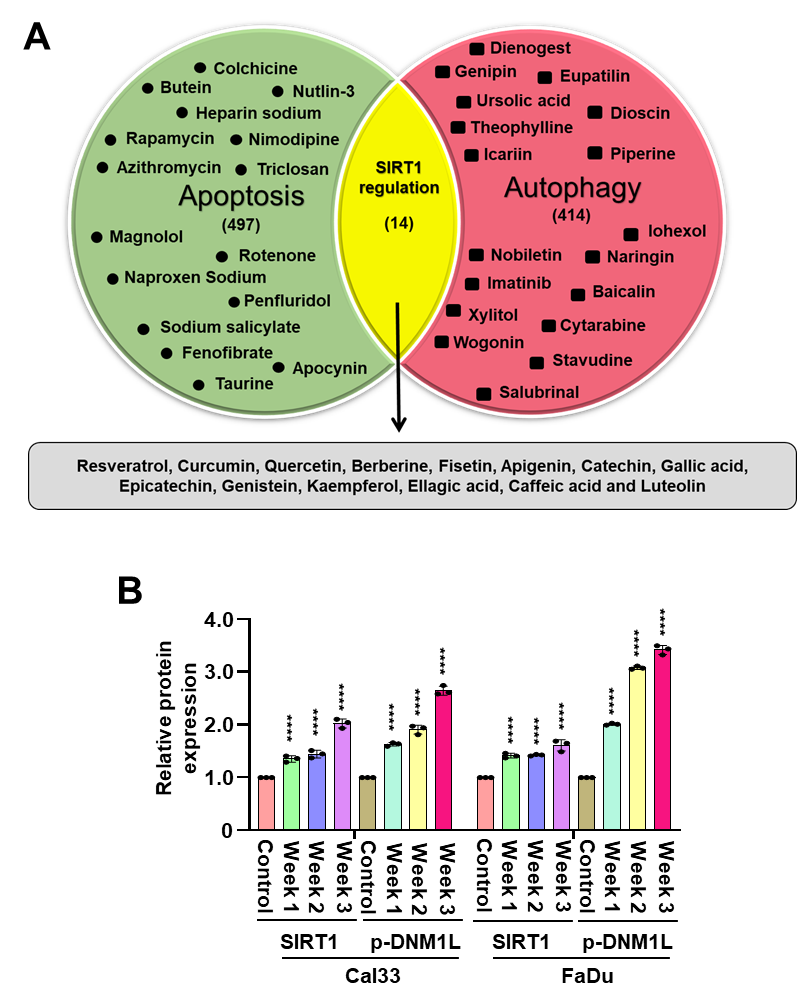


**Supplementary Fig. S5.** To find a novel activator of SIRT1 that can further be used as an anticancer drug, we were keen to screen various compound library and online database for apoptosis and autophagic cell death inducing compounds. A total of 621 compounds were primarily identified as targetable pharmacophores. The secondary screening identified 497 apoptosis and 414 autophagy targeting compounds that positively regulate these cell death pathways. Further screening for identification of common death pathway regulators demonstrated 16 compounds that has SIRT1 activating potential (S5A). The confirmation of best targetable compound was done by evaluating the pharmacokinetics and pharmacodynamics by SWISS ADME data base. The quantification of western blots such as SIRT1 and p-DNM1L was represented (S5B).

**Supplementary Figure 6 (S6)**

**
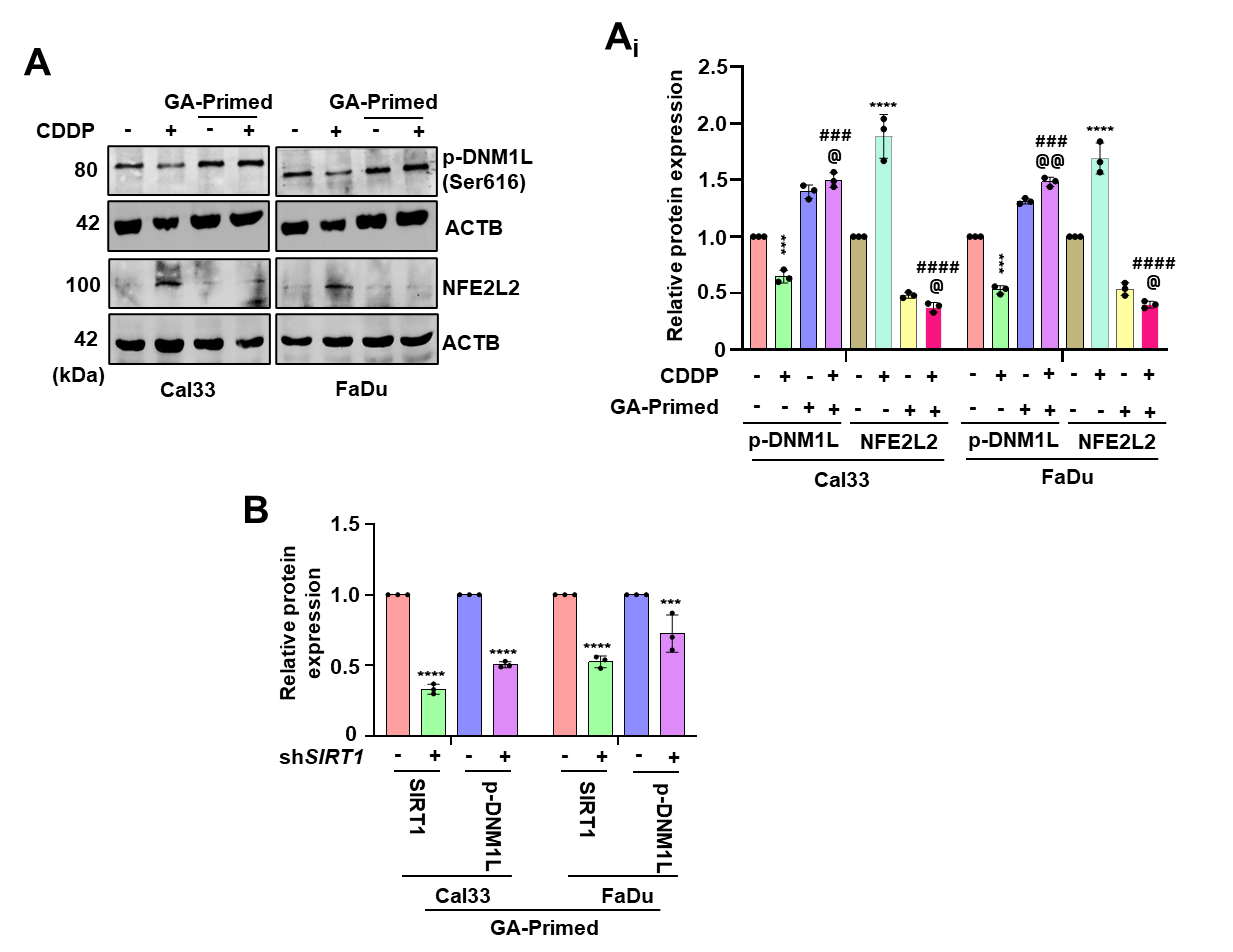
**

**Supplementary Fig. S6.** The expression of p-DNM1L and NFE2L2 were represented in the CDDP treated GA-primed cells (S6A-A_i_). The quantification of western blots such as SIRT1 and p-DNM1L was represented (S6B).

**Supplementary Figure 7 (S7)**


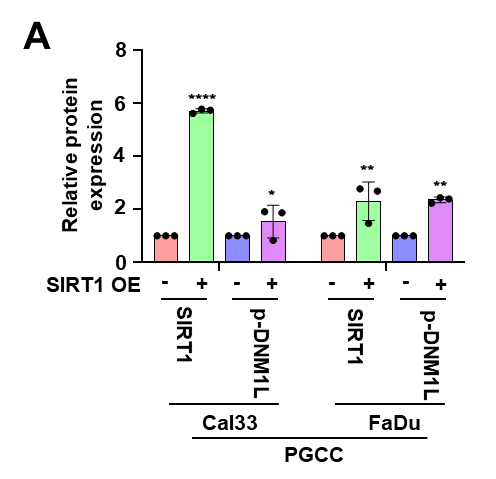


**Supplementary Fig. S7.** The quantification of western blots such as SIRT1 and p-DNM1L was represented (S7A).

**Supplementary Figure 8 (S8)**


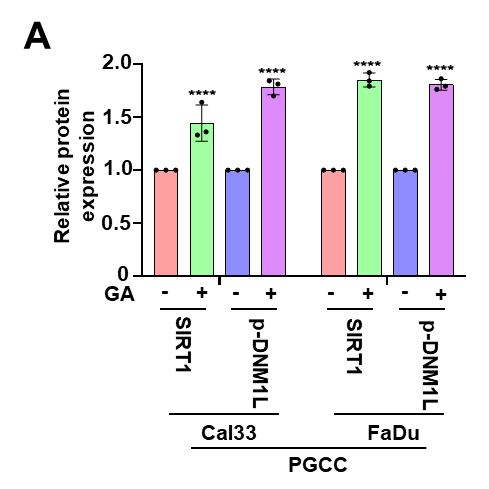


**Supplementary Fig. S8.** The quantification of western blots such as SIRT1 and p-DNM1L was represented (S8A).
