# Supplementary material for: SIRT1 inhibits mitochondrial hyperfusion associated mito-bulb formation to sensitize oral cancer cells for apoptosis in a mtROS-dependent signalling pathway
Source: Cell Death Dis. 2023 Nov 10;14(11):732. doi: 10.1038/s41419-023-06232-x (PMC10638388; doi:10.1038/s41419-023-06232-x)

Figure 1B

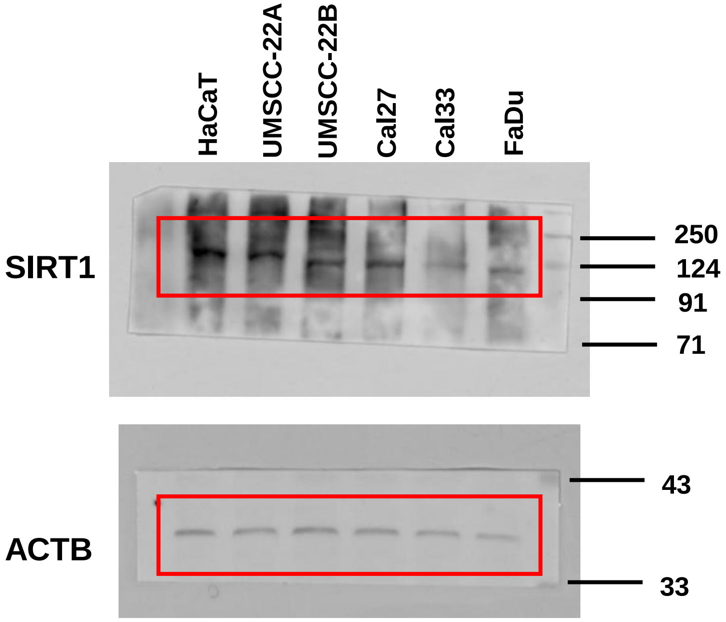

Figure 1E

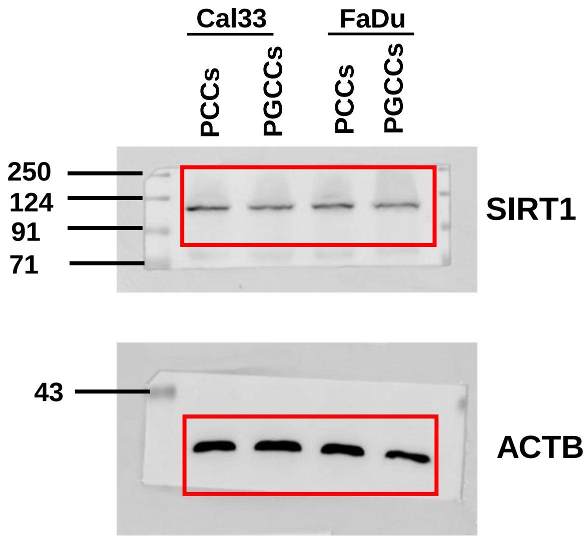

Figure 2A

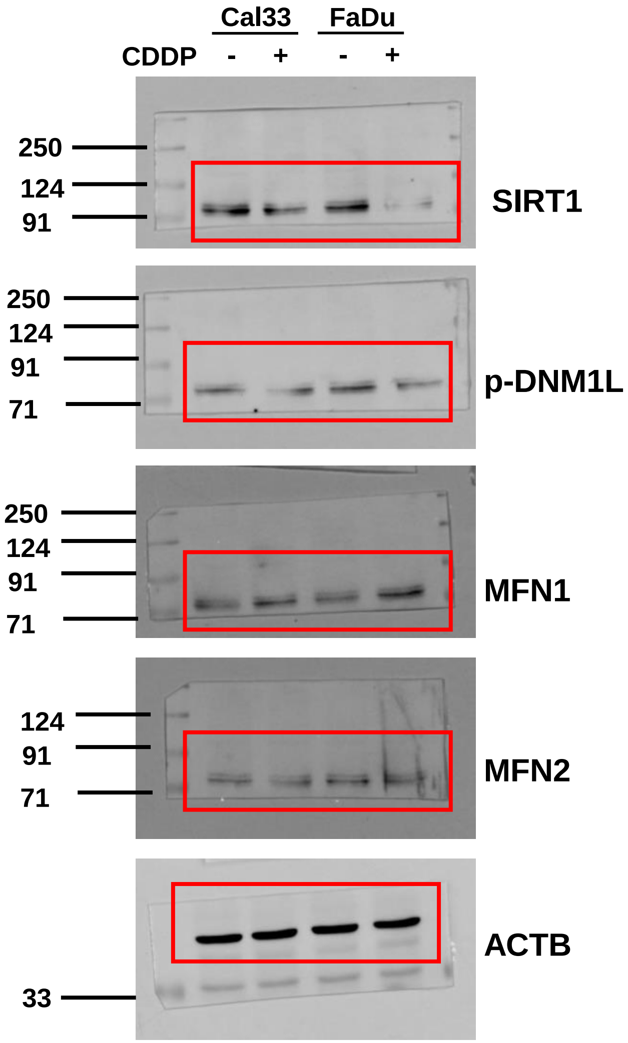

Figure 2Ci

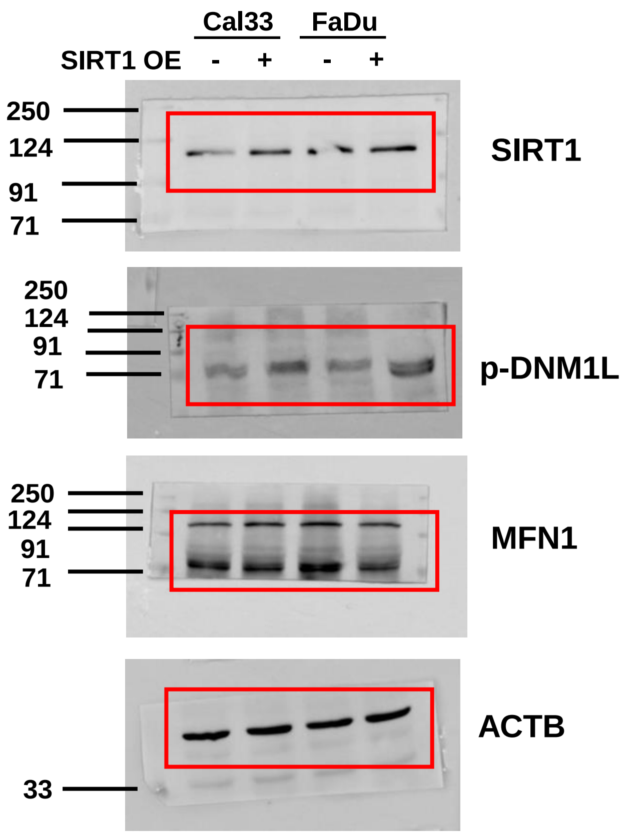

Figure 2Cii

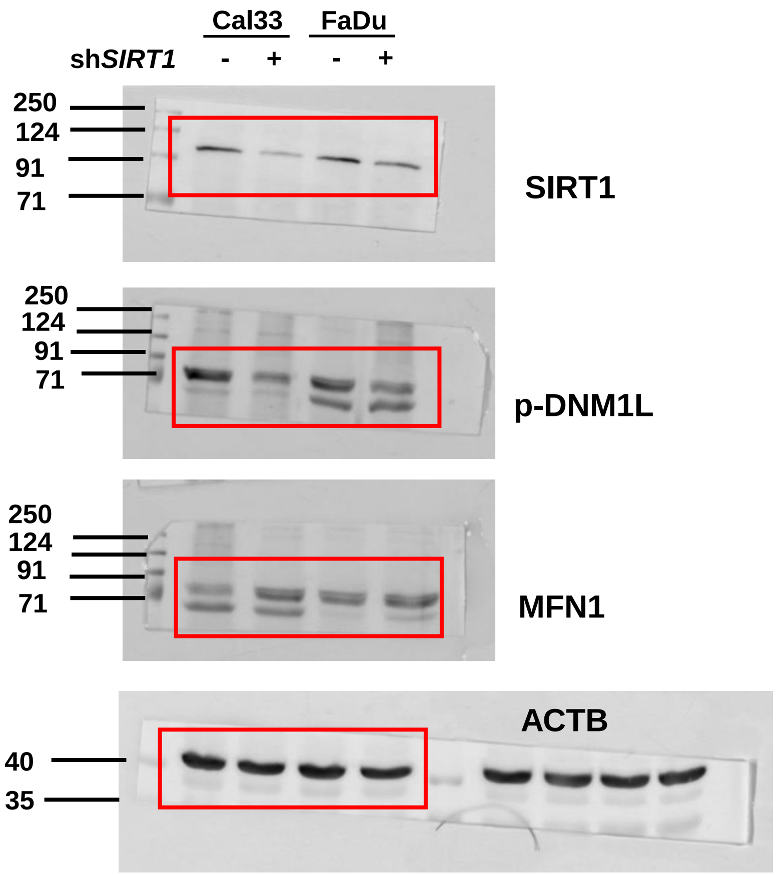

Figure 5A

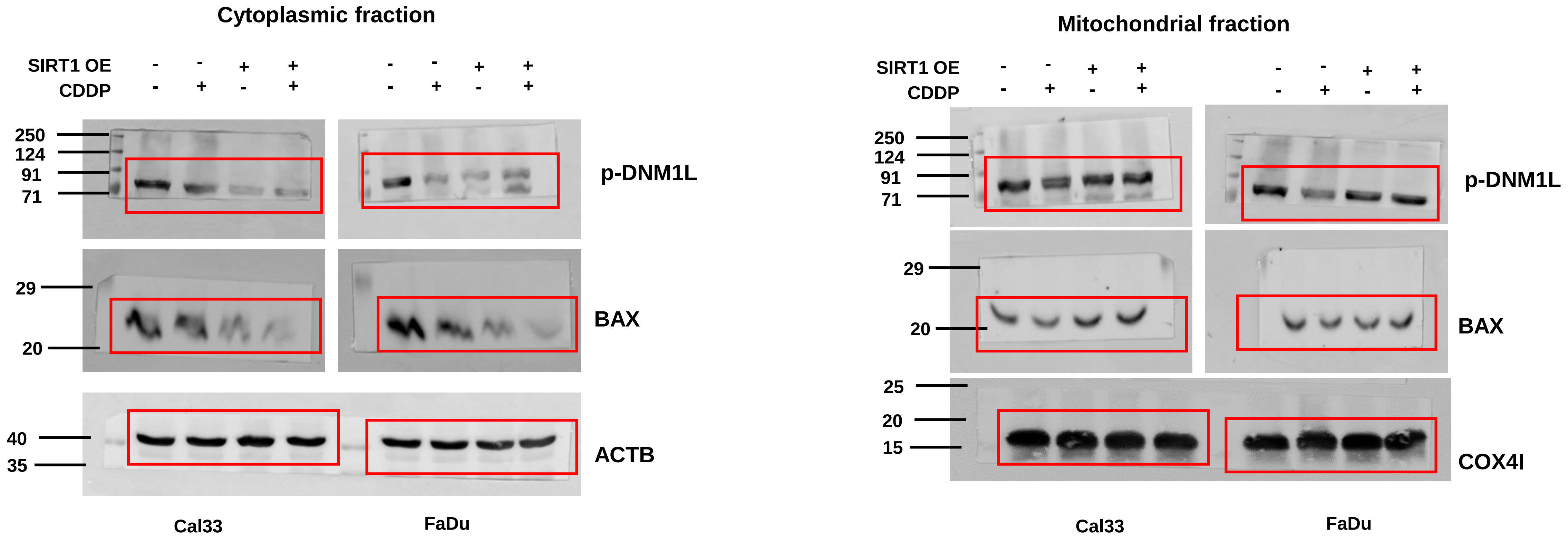
Cytoplasmic fraction
Mitochondrial fraction
SIRT1 OE
CDDP
- - + + - - + +
- + - + - + - +
250
124
91
71
p-DNM1L
29
20
BAX
40
35
ACTB
25
15
COX4I
Cal33
FaDu

Figure 6A

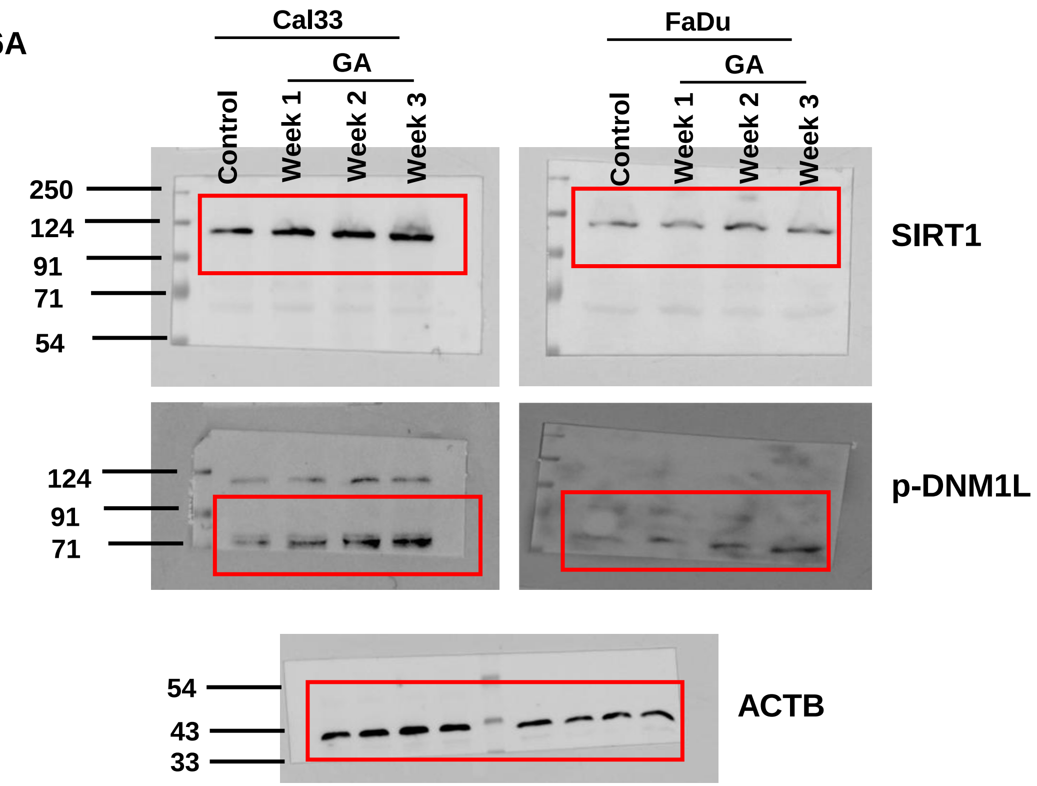

Cal33
GA
Control
Week 1
Week 2
Week 3
FaDu
GA
Control
Week 1
Week 2
Week 3
250
124
91
71
54
SIRT1
124
91
71
p-DNM1L
54
43
33
ACTB

Figure 7A

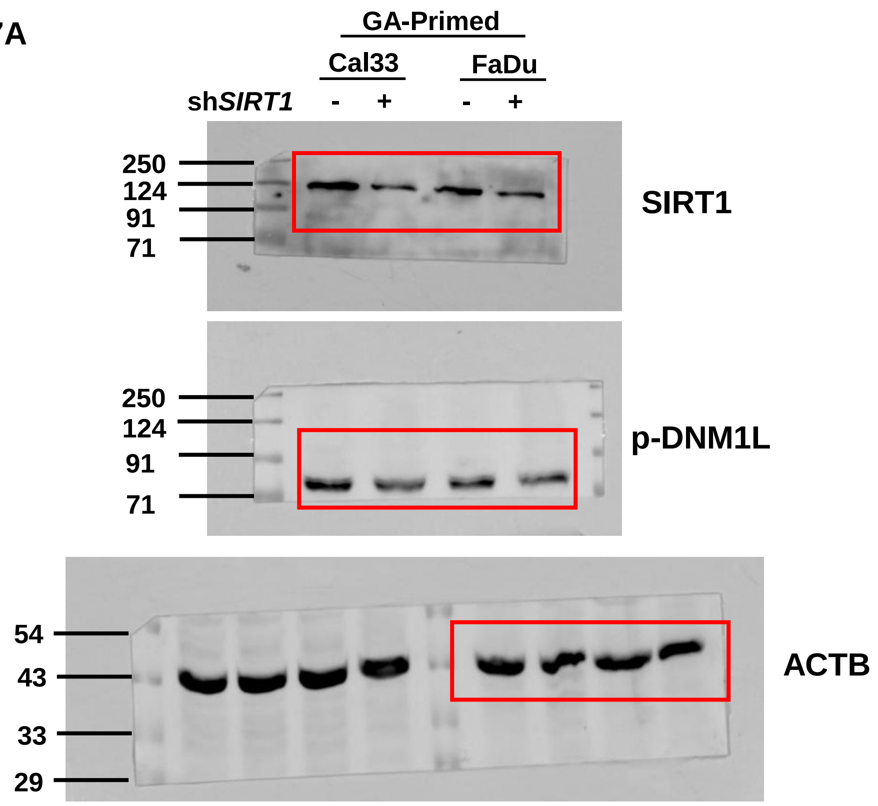

GA-Primed
Cal33
FaDu
shSIRT1
-
+
-
+
250
124
91
71
SIRT1
250
124
91
71
p-DNM1L
54
43
33
29
ACTB

Figure 8A

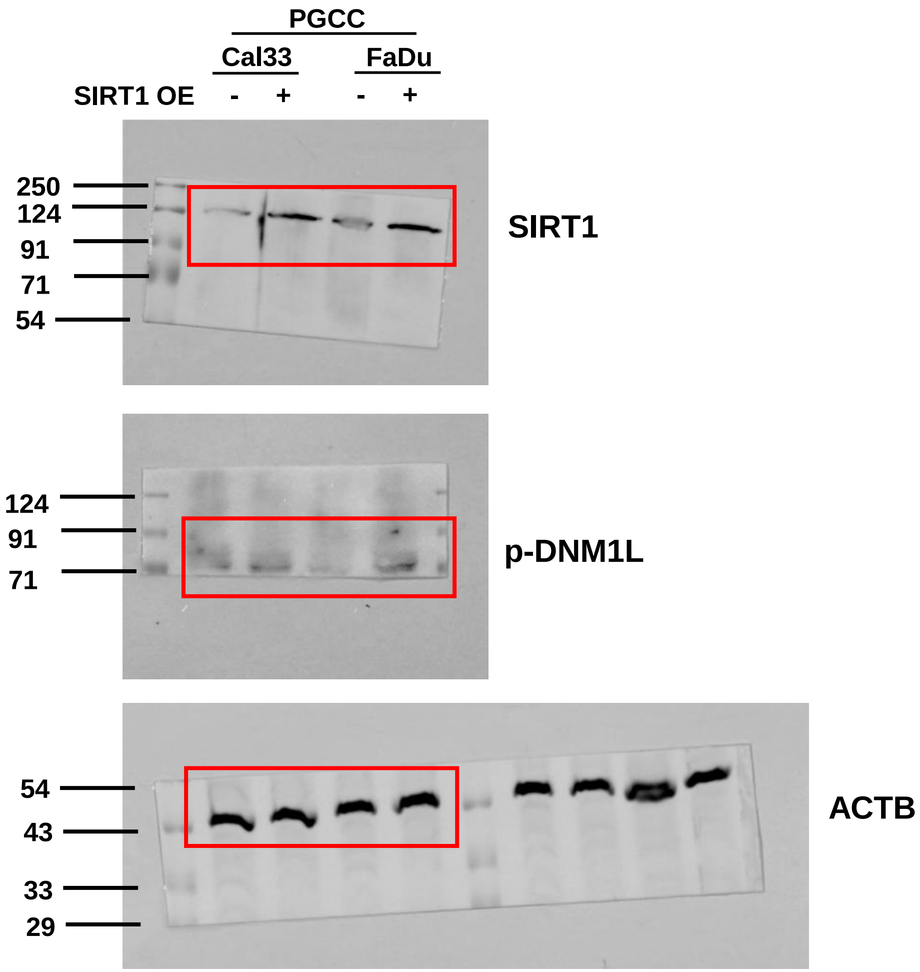
PGCC
Cal33
FaDu
SIRT1 OE
-
+
-
+
250
124
91
71
54
SIRT1
124
91
71
p-DNM1L
54
43
33
29
ACTB

Figure 9A

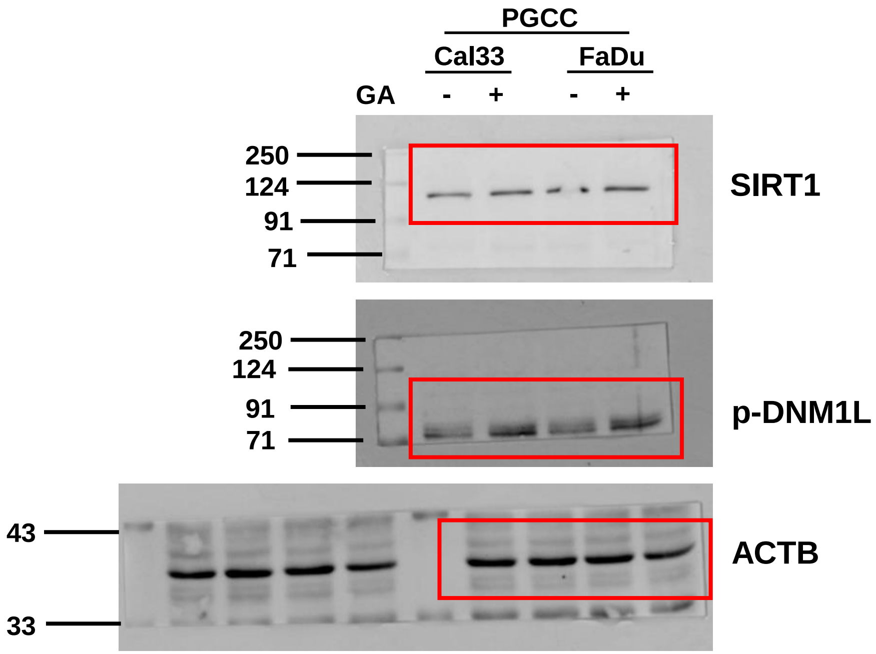
PGCC
Cal33
FaDu
GA
-
+
-
+
250
124
91
71
SIRT1
250
124
91
71
p-DNM1L
43
33
ACTB

Supplementary figure S2D

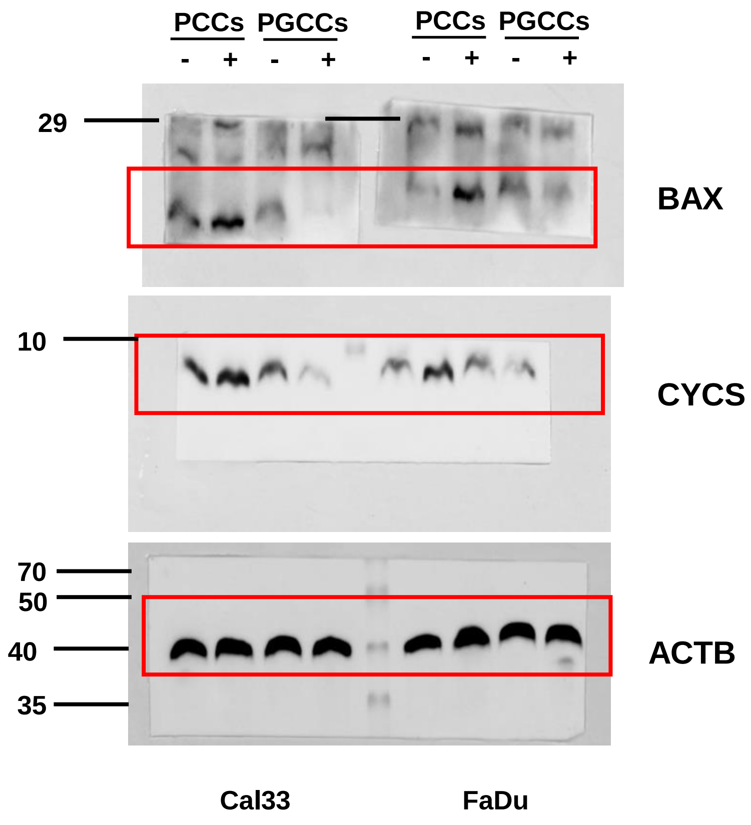

Supplementary figure S4

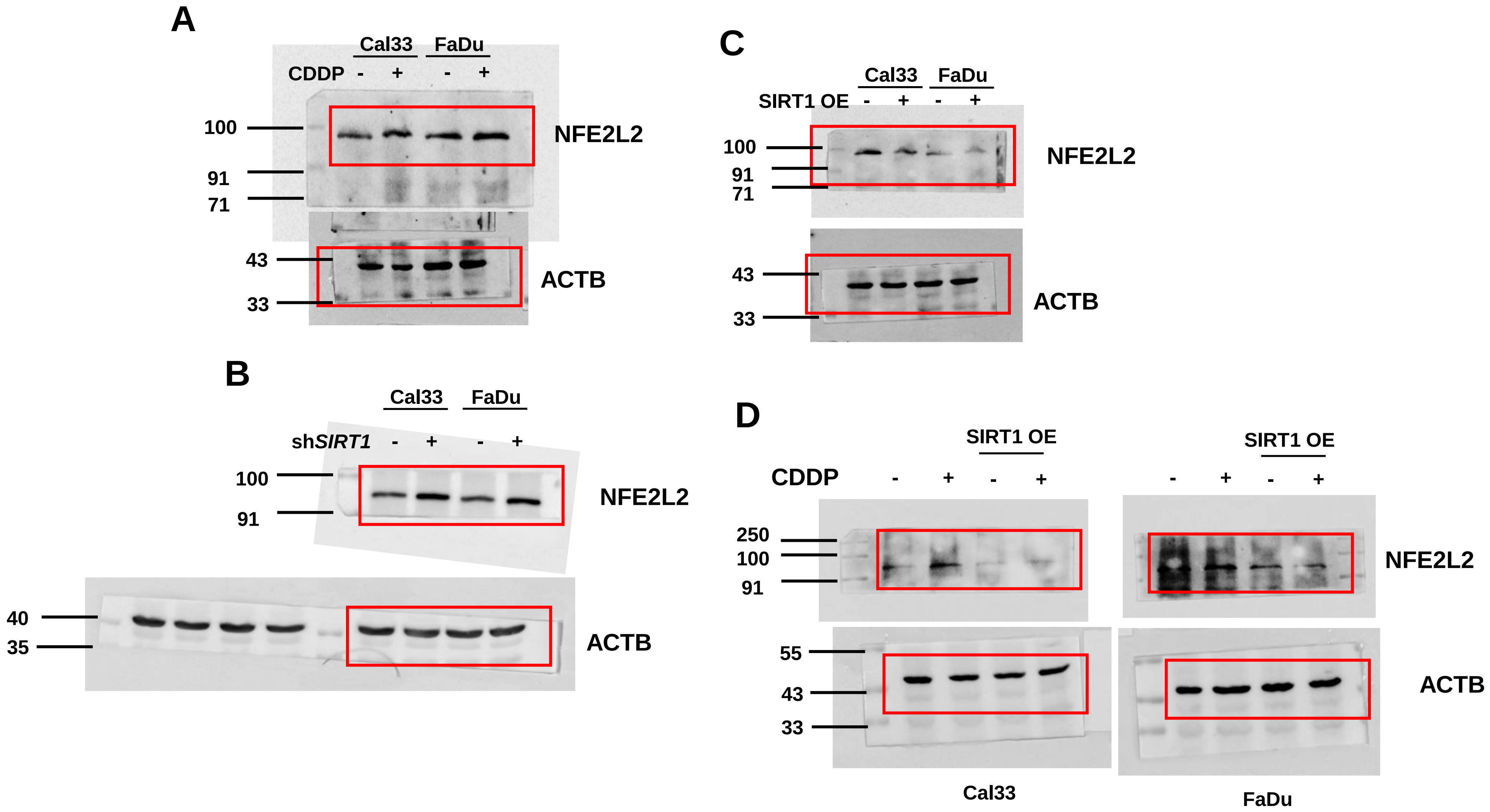

Supplementary figure S6

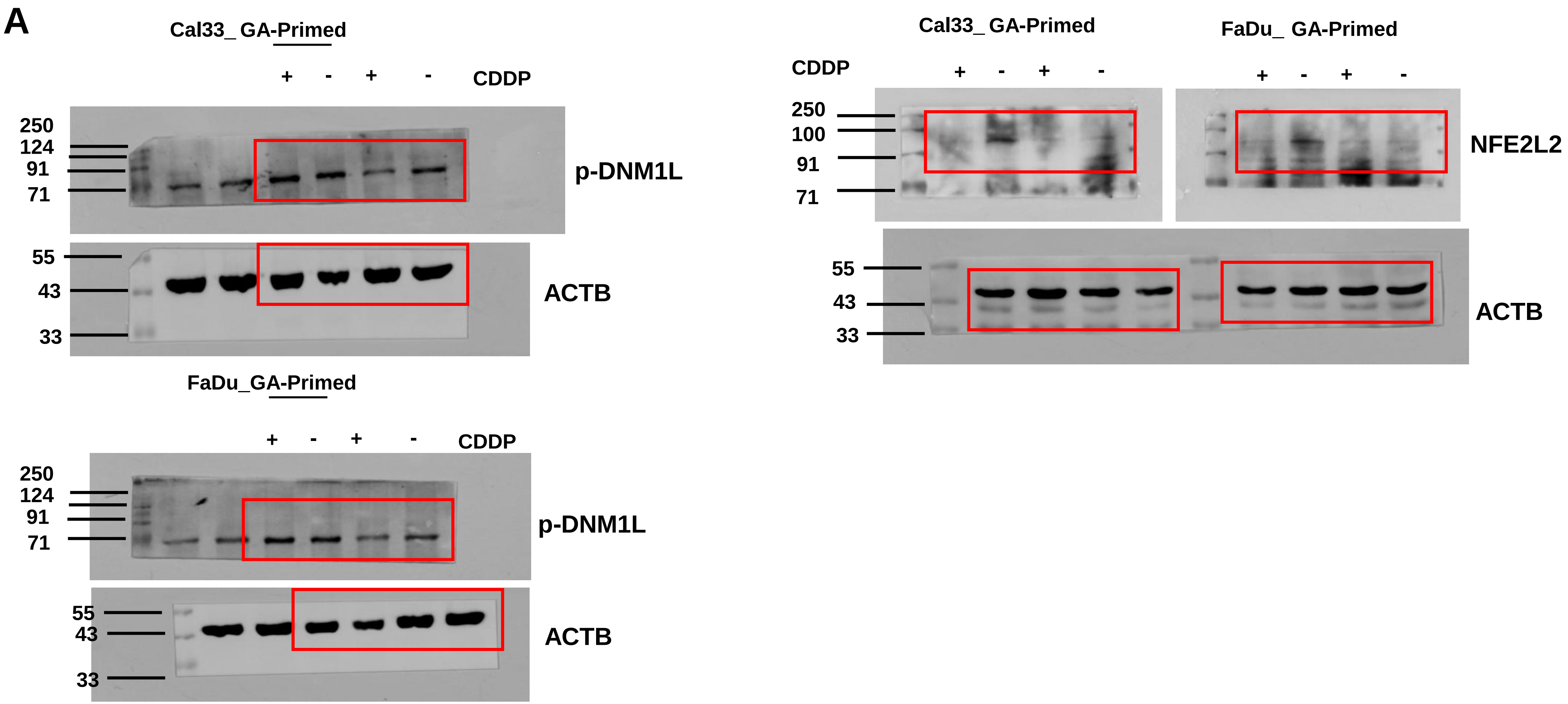

Supplement: Supplementary file 2 — Original Data File [file 41419_2023_6232_MOESM2_ESM.pdf]
